# Impact of Changes in Buffer Ionic Concentration and Mutations on a GH1 β‑Glucosidase Homodimer

**DOI:** 10.1021/acsomega.5c03396

**Published:** 2025-08-07

**Authors:** Rafael S. Chagas, Sandro R. Marana

**Affiliations:** Departamento de Bioquímica, Instituto de Química, 28133 Universidade de São Paulo, São Paulo 05508-220, Brazil

## Abstract

Oligomerization is a key feature of protein function, with approximately 30% of proteins exhibiting this trait. The homodimeric form of proteins, such as the GH1 β-glucosidase from *Spodoptera frugiperda* (Sfβgly), plays a significant role in enzyme activity. In this study, we investigate the homodimerization of Sfβgly, which forms a cyclic C2 dimer with a well-defined interface. Using size exclusion chromatography and SEC-MALS, we characterized the homodimerization behavior of Sfβgly at equilibrium conditions in different ionic concentrations of phosphate buffer. The dissociation constants (*K*_D_) increase with decreasing ionic concentration, suggesting that the hydrophobic effect is central to homodimer formation. Site-directed mutagenesis of key residues at the dimer interface further elucidated the contributions of specific amino acid residues to dimer stability. Mutations affecting both, apolar and hydrogen bond-forming residues, significantly increased the *K*_D_. However, mutations of hydrogen bond-forming residues caused a smaller *K*_D_ change than apolar residue mutations, suggesting that while the latter is the driving factor in the dimerization, the former could play a role in guiding the monomers relative orientation. These findings enhance our understanding of protein oligomerization in GH1 β-glucosidases and its implications for protein design and function.

## Graphical abstract

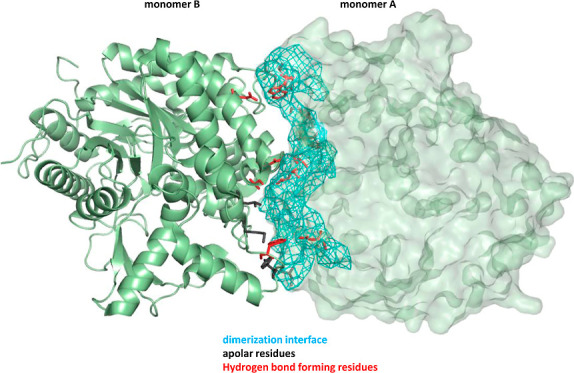

## Introduction

Oligomerization is a well-documented phenomenon in proteins, crucial for their biological function. This process occurs in all life forms and involves approximately 30% of all the proteins.^1,2^ Several significant proteins such as enzymes active upon DNA,^3^ tyrosine kinase receptors,^4^ G-protein-coupled receptors,^5^ transcriptional factors,^6^ and ion channels^7^ are oligomers.

Oligomeric proteins are composed of multiple subunits (polypeptide chains), which may be identical (a homo-oligomeric protein) or different (hetero-oligomers).^8^ The term homomer has been commonly used instead of homo-oligomer to refer to a protein oligomer formed by self-interacting copies of a protein subunit.^1^ The most frequent forms of homomers are dimers and tetramers, which occur approximately four times more often than heterooligomers.^9^ Nevertheless, the determination of the prevalence of hetero-oligomers under intracellular conditions is not easily accessible.

The formation of homomers provides several structural and functional benefits,^10^ including enhanced stability,^11,12^ control over the accessibility and specificity of active sites,^3^ and structural complexity. It also minimizes genome size^13^ because a protein that exists as a dimer requires only a single encoding sequence in the genome, unlike a monomeric protein of the same molecular weight. This concept is referred to as “genetic saving”.^14^

In the UniProt Database, the most comprehensive protein database, homodimeric proteins make up over 51%, significantly surpassing the proportion of monomeric and other oligomeric forms.^14^ According to the BRENDA enzyme database,^15^ the majority of enzymes are homomers, with dimers being more prevalent than monomeric enzymes.^15,16^ Here, we investigated the homodimer of the GH1 β-glucosidase from *Spodoptera frugiperda* (Sfβgly), a secreted digestive enzyme associated with the glycocalyx of midgut epithelial cells in the cited armyworm.^17^ This enzyme has been extensively studied regarding its catalysis, thermal stability, substrate recognition, and oligomerization.^17–20^ General features for GH1 β-glucosidases, enzymes grouped based on their sequence,^21^ include the TIM barrel ([β/α]_8_ barrel) fold, the hydrolysis of *O*- or *S*-glycosidic linkages, and a tendency to form homomers. However, the regions involved in the formation of the oligomerization interface vary from one enzyme to the other,^22^ reflecting the diversity of their quaternary structures.^23^

Sfβgly (5CG0 PDB code) exhibits a cyclic symmetry, specifically a C2 homodimer, which means that rotating the complex by 180° around the symmetry axis results in the subunits being mutually superposed.^24^ In this type of symmetry, the contact between subunits is made by the same surface in both monomers, which is referred to as an isologous interface.^25^ The dimeric interface in Sfβgly encompasses 905 Å^2^, 5% of the total surface area in each monomer. It is composed of 30 residues, 63% of which are apolar, and 4 residues in each monomer are involved in hydrogen-bond formation. The interface presents no disulfide bond or salt bridges (Figure 1 and Table 1). As seen, the interface comprises four short segments of contiguous residues: T108 to N112, K148 to R169, Y196 to N218 and Q301 to W305 (Table 1), which are respectively placed at the loop_2_, loop_3_-α-helix_3_, loop_4_-α-helix_4_ and loop_5_ of the (β/α)_8_ barrel fold of the Sfβgly.

**Figure 1:**
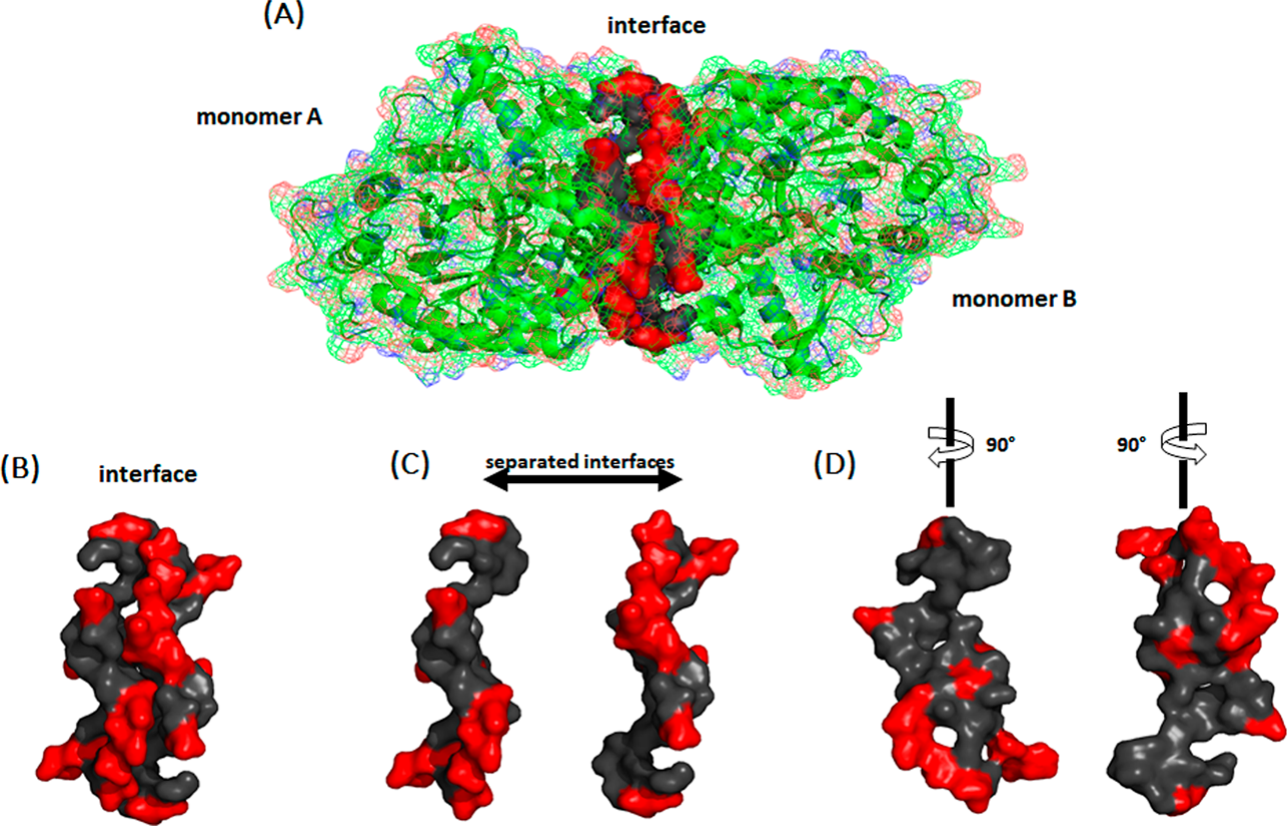
Sfβgly homodimer (PDB 5CG0). (A) Interface residues are represented as a solid molecular surface (gray and red), whereas the two monomers are shown as cartoons covered with a wire superficial envelope. Apolar residues are in gray and polar residues are in red. (B) Dimerization interface surface. (C) Separated dimerization interface surface. (D) Both surfaces were rotated 90°.

**Table 1:** Residues Forming the Sfβgly Homodimer Interface^a^

residue	relative surface area buried in the interface (%)	residue	relative surface area buried in the interface (%)
T108	10	R169	10
**M110**	**80**	**Y196**	**20**
**A111**	**70**	**L205**	**70**
N112	80	N206	10
K148	10	**A207**	**40**
Q150	20	**M210**	**70**
E151	60	**G211**	**80**
**L152**	**90**	**L214**	**70**
**G153**	**100**	N218	10
A156*	**100**	Q301	30
N157*	100	**G302**	**40**
**P158**	**60**	**Y303**	**80**
**L159**	**60**	**P304**	**30**
**W163**	**60**	W305*	**40**
D166*	80	**P348**	**10**

a The lines occupied by apolar residues are marked in bold. Residues involved in hydrogen bonds are marked with an asterisk. Relative surface area buried in the interface corresponds to the ratio between the total surface area accessible to the solvent in the monomer and the surface area buried in the dimer interface. Interface residues were identified by using the PDBePISA based on the crystallographic structure 5CG0.

Oligomerization in GH1 β-glucosidases has been hypothesized as an adaptive strategy for thermal stability.^26,27^ Also, it was demonstrated for Sfβgly that oligomerization turns the homodimer more active than the monomer and that the oligomerization occurs through a conformational selection mechanism, i.e., monomers exchange between two conformations in equilibrium and only one of them combines with a similar one, forming the homodimer.^20^

The phenomenon of protein oligomerization can be modulated by protein concentration, ionic strength, pH, effectors and nucleic acids while examples of artificial modulators are cross-linkers, shiftides and nanoparticles.^2^ In the present study, we investigated the Sfβgly homodimer formation in a range of different concentrations of phosphate buffer, which made it possible for us to estimate the homodimer dissociation constant (*K*_D_) in different ionic strengths. We also introduced simple mutations directed to the homodimer interface residues, which contribute to suggesting their individual contributions to the homodimerization. The results showed that the hydrophobic effect is the main component that leads to dimerization in Sfβgly, whereas residues involved in hydrogen bond formation probably act as guidance to the monomers relative orientation.

## Results

### Characterizing the Dimerization of Sfβgly in Different Ionic Concentration Buffers

The wild-type Sfβgly was purified and its purity was verified using SDS-PAGE (Figure S1). Its oligomeric states were assessed through size exclusion chromatography (SEC) in different concentrations of the sodium phosphate buffer. Sfβgly appeared as both a monomer (retention volume ≈35 mL) and a dimer (retention volume ≈25 mL) in 100 mM sodium phosphate buffer pH 6 (P100). Their identity was assessed and confirmed by SEC-MALS (Figure 2), confirming a previous observation.^20^

**Figure 2:**
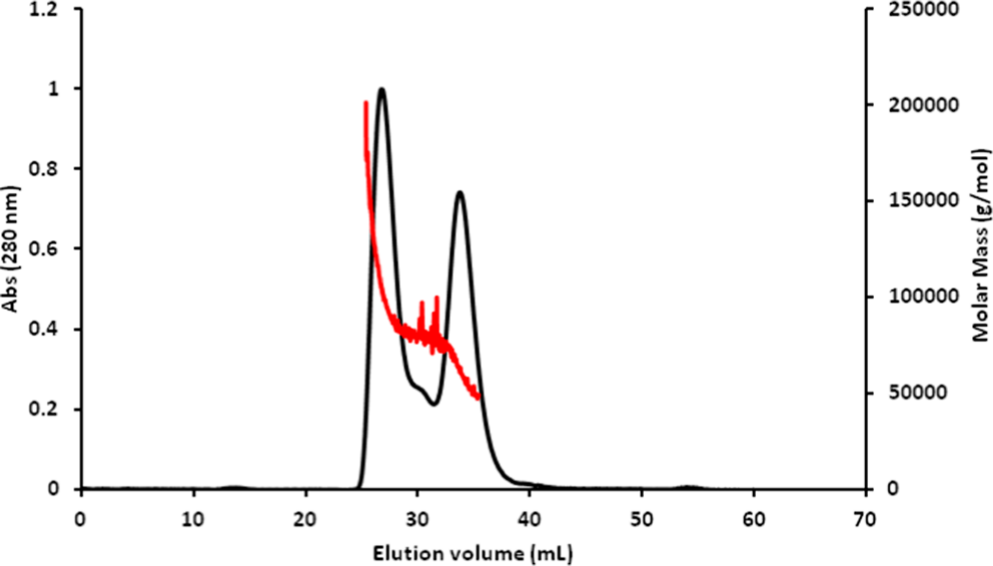
Analysis of the purified wild-type Sfβgly in size exclusion chromatography coupled to a multiple-angle light scattering detector (SEC-MALS). Experiment performed with 90 μM Sfβgly in P100 at 5 °C.

The same oligomeric states were observed in 75 mM, 50 mM, 25 mM, and 5 mM sodium phosphate buffers pH 6 (P75, P50, P25, and P5, respectively) (Figure 3).

**Figure 3:**
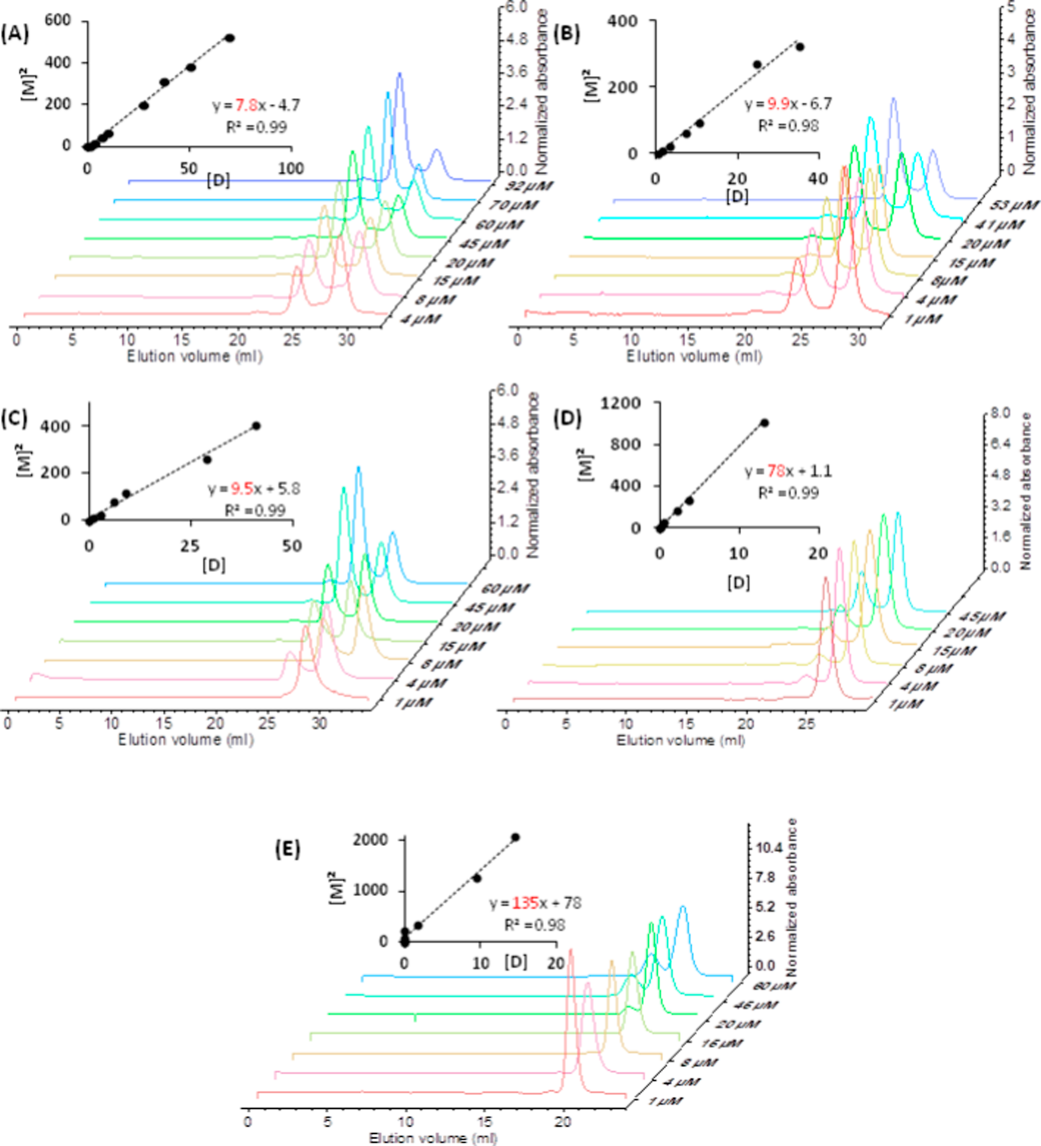
Estimation of the *K*_D_ of the Sfβgly homodimer at different ionic concentrations. (A) P100; (B) P75; (C) P50; (D) P25; (E) P5. [M] stands for monomer concentration. [D] is the homodimer concentration. Line slopes, which correspond to the *K*_D_, are shown in red. Experiments were conducted at pH 6 at 5 °C. The experiment with the wild-type Sfβgly at P100 was repeated with three independent samples (Figure S2). See Materials and Methods for details.

In the buffer concentrations tested, the balance between the homodimer and monomer, deduced based on the ratio of their chromatographic peaks, changed as different concentrations of Sfβgly were examined (Figure 3). In P100, at higher Sfβgly concentrations, the dimeric state predominated. But, as the Sfβgly concentration decreased, the balance gradually reversed, with the monomeric state becoming more prevalent. These observations confirm that the homodimer formation is a reversible process,^20^ which can be studied by assuming a simple equilibrium M + M ⇌ D. Interestingly, as the buffer concentration decreased, a higher concentration of Sfβgly was required to shift the equilibrium toward the dimer prevalence. That reflects changes in the dimer dissociation constant (*K*_D_).

Note that the SEC was performed at 5 °C to ensure a slow rate of monomer–dimer interconversion. Hence their populations remain in equilibrium even during the chromatographic separation. The experiment with the wild-type Sfβgly at P100 was repeated with three independent samples to ensure the reproducibility of the procedure and also to estimate the experimental deviation of the *K*_D_ under these conditions (Figure S2). Based on that, the *K*_D_ for the wild-type Sfβgly dimer at P100 is 7 ± 1 μM, which corresponds to a small relative deviation (14%).

To assess the effect of the ionic concentration on the dimerization process, we determined the *K*_D_ based on the area of the chromatographic peaks (Figure 3).

Thus, the homodimer *K*_D_ remained stable at P100, P75, and P50, *K*_D_ = 7, 9.9, and 9.5 μM, respectively, but it presented pronounced changes at P25 and P5, *K*_D_ = 78 and 135 μM, respectively. It is noteworthy that the *K*_D_ changes at P25 and P5 are beyond the estimated experimental deviation observed with the wild-type protein (14%). For generalization purposes, the *K*_D_ was also expressed as a function of the molar ionic strength (I) resulting from the buffer concentrations (Figure 4).

**Figure 4:**
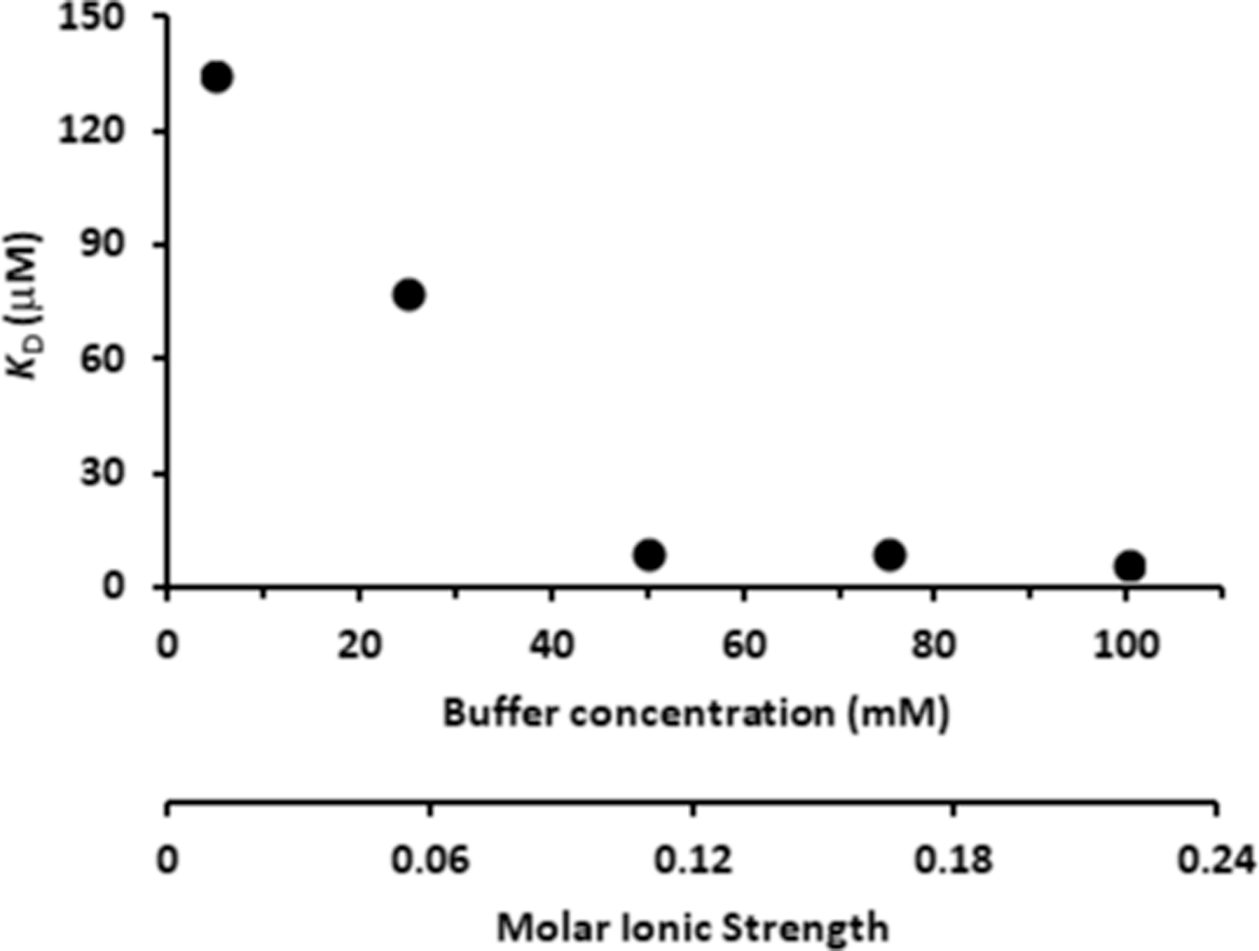
Effect of the phosphate buffer concentration and the corresponding ionic strength on the dissociation constant (*K*_D_) of the Sfβgly dimer. The different ionic strength results from changes in the phosphate buffer concentration (P100, *I* = 0.218; P75, *I* = 0.163; P50, *I* = 0.109; P25, *I* = 0.054; P5, 0.011). The experimental deviation of the *K*_D_ at P100 is 14%, which may also apply to the different buffer concentrations.

In summary, the increment in the concentration of the phosphate buffer, i.e., ionic strength, resulted in a starkingly decrease of the *K*_D_ (Figure 4), indicating a drastic increase in the affinity between the Sfβgly monomers. A similar effect is observed in hydrophobic interaction chromatography, in which a high salt concentration promotes the interaction between apolar molecules. Conversely, if the monomer binding were mainly driven by noncovalent interactions based on electric charges (ionic bridges and hydrogen bonds), the rise in the ionic concentration would hamper the dimerization, i.e., increase the *K*_D_. Hence, Figure 4 suggests that the hydrophobic effect is the main component impelling the Sfβgly dimerization.

### Detailing the Dimerization of Mutant Sfβgly

Previous experiments provided a broad perspective of the forces guiding the Sfβgly dimerization. However, individual residues of the dimerization interface may have different relative contributions to that process. Aiming at to uncover those roles, point mutations were introduced at residues presenting more than 70% of their surface area buried in the interface (Table 1). Residues involved in hydrogen bonds were also included regardless their buried surface area. The A and G residues were not targeted due their small side-chains, which corresponds to small areas even in the case of a higher percentage of buried surfaces. Thus, six different mutant Sfβgly, each of them with a single replacement (N112S, N157S, D166S, M210A, L214A, and Y303A), were expressed as folded soluble proteins in bacteria and purified (Figures S1 and S3).

The *K*_D_ of the mutant Sfβgly homodimers was evaluated by the same methodology employed for the wild-type Sfβgly (Figure 5, and Table 2).

**Figure 5:**
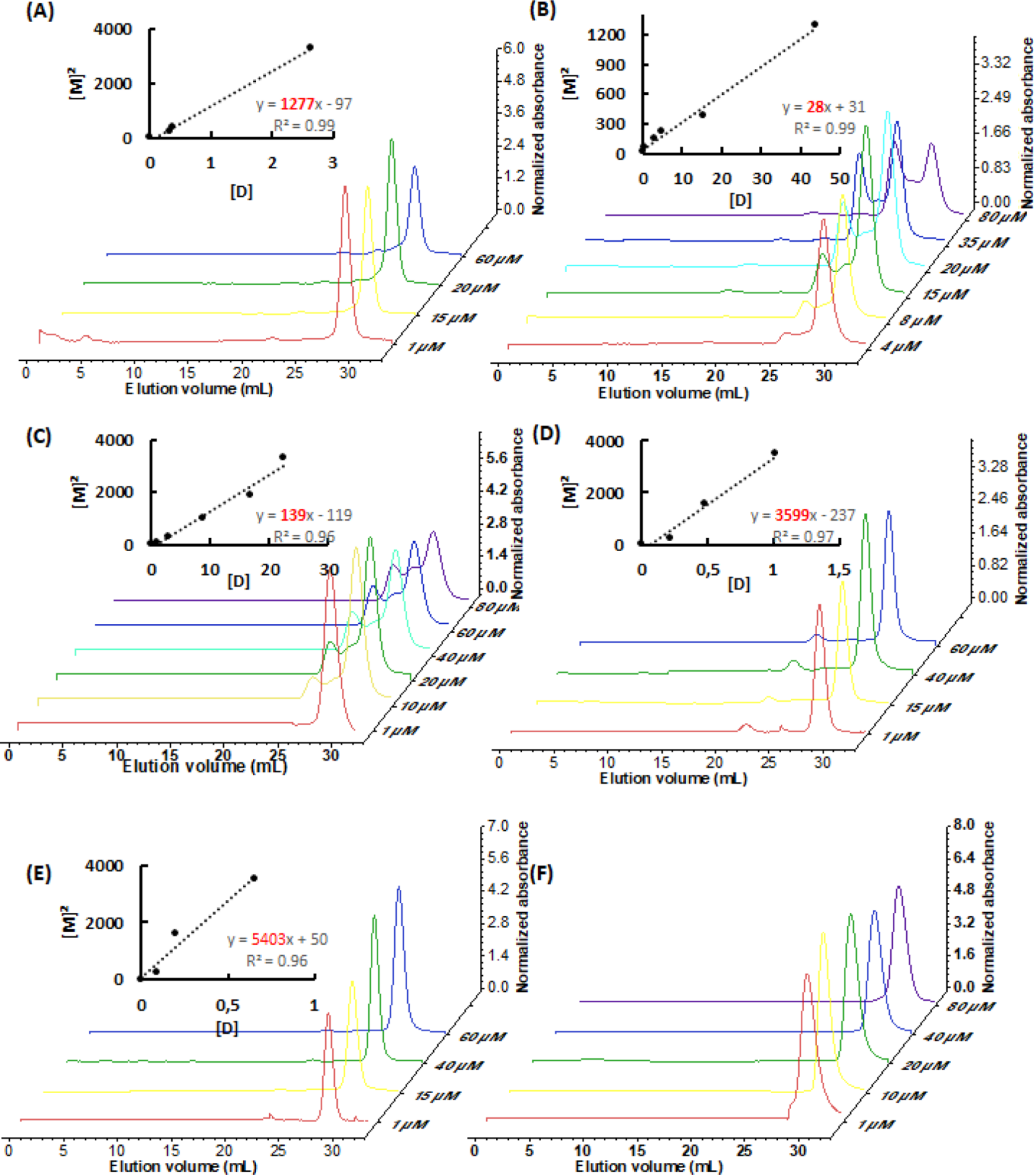
Estimation of the *K*_D_ of the mutant Sfβgly homodimer in P100. (A) N112S, (B) N157S*, (C) D166S*, (D) M210A, (E) L214A and (F) Y303A. Mutant Y303A was observed as a monomer. Asterisks indicate mutations of residues involved in the hydrogen bond between the monomers in the interface of the wild-type protein dimer.

**Table 2:** *K*_D_ and Free Energy Change (ΔΔ*G*_diss_^0^) of the Homodimer Dissociation for the Wild-Type and Mutant Sfβgly^a^

Sfβgly	*K*_D_ (mM)	ΔΔ*G*_diss_^0^ (kJ/mol)
wild-type	0.008	
N112S	1.2	12
N157S*	0.028	2.9
D166S*	0.1	6.6
M210A	3.6	14
L214A	5.4	15
Y303A	nd	nd

a Nd, not determinable. Mutant Y303A was observed as a monomer. So, for mutant Y303A the *K*_D_ and ΔΔ*G*_diss_^0^ must be higher than the highest observed, i.e. >5.4 mM and >15 kJ/mol. ΔΔ*G*_diss_^0^ were calculated at 5 °C, the same temperature used in the SEC. Asterisks indicate mutations of residues involved in a hydrogen bond between the monomers in the interface of the wild-type homodimer. The *K*_D_ experimental deviation is 14% based on the experiments conducted with the wild-type enzyme (Figures 3 and S2).

### Residues Involved in the Hydrophobic Effect

The single mutations N112S, M210A, and L214A were sufficient to drastically change the *K*_D_ of the dimer, causing an increase of 3 orders of magnitude. Mutant Y303A was found only in the monomeric form, suggesting an even more drastic increase in the *K*_D_. Based on the relationship between *K*_D_ and Δ*G*_dissociation_^0^, ΔΔ*G*_diss_^0^ = *RT* ln­(*K*_Dwild‑type_/*K*_Dmutant_), the average loss in affinity between the monomers is approximately 14 *k*J/mol (Table 2), which corresponds to about 50% of the binding energy between the monomers (29 *k*J/mol; based on the *K*_D_ of the wild-type Sfβgly dimer). The loss of affinity resulting from the Y303A mutation is even more significant, certainly surpassing the above values and preventing dimer formation.

### Residues Involved in the Hydrogen Bonds

Moving forward, we also tested the N157S and D166S mutants, which allowed an estimation of the hydrogen bond energy at the interface of the Sfβgly homodimer. There are four hydrogen bonds between the monomers in the dimer, N157 acting as a donor in the interaction with A156 and the residue D166 as an acceptor in the interaction with W305 (Figure 6). Thus, each monomer has 2 donors and 2 acceptors involved in the formation of the four hydrogen bonds. Therefore, just two mutations, N157S and D166S, are enough to evaluate the role of the four hydrogen bonds at the homodimer interface. Notably, there is one hydrogen bond at each edge of the interface and two in the center, hence the spatial distribution of the donors and acceptors allows only one relative orientation of the monomers in which all four bonds are formed (Figure 6).

**Figure 6:**
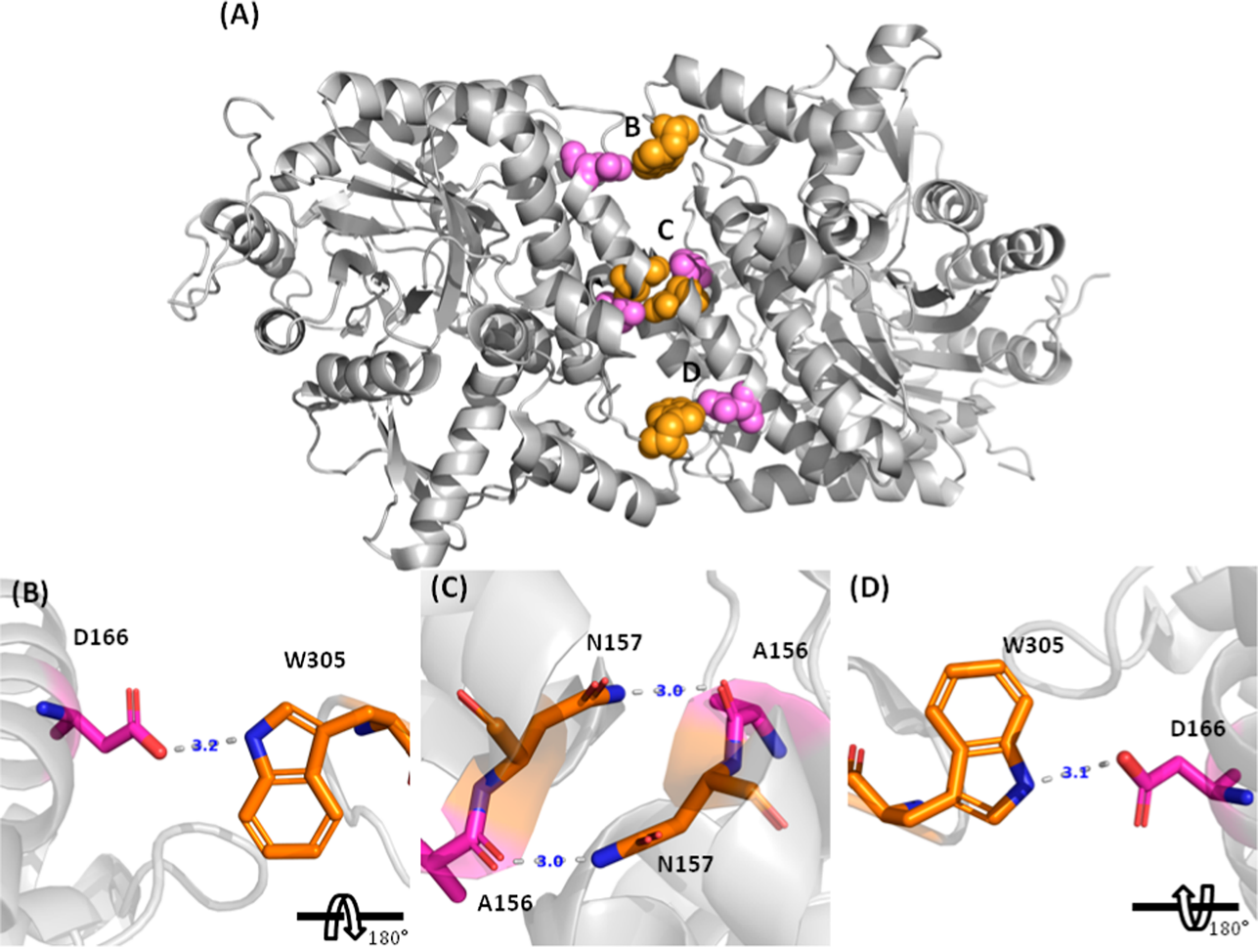
Hydrogen bonds at the Sfβgly homodimer interface (PDB 5CG0). (A) Residues in pink are acceptors, while those in orange are donors. (B–D) panels detail the hydrogen bonds (dashed lines) and their positions at the interface. Distances (in blue) are in Å. The letters are the same as in panel (A): (B) and (D) indicate interactions at the interface extremities and (C) indicates interactions at the center.

The hydrogen bond between N157 and A156 corresponds to 2.9 kJ/mol, while the bond involving D166 and W305 corresponds to 6.6 kJ/mol (Table 2). These values are typical of hydrogen bonds in proteins.^28^ Moreover, they indicate that, collectively, the four hydrogen bonds contribute with 19 kJ/mol (2 × 2.9 + 2 × 6.6) to the stabilization of the Sfβgly homodimer.

### Evaluating the Conservation of the Dimerization Interface

Next, we aimed at to evaluate if the principles guiding the Sfβgly dimerization, hydrophobic effect and hydrogen bonds, are shared among the GH1 β-glucosidases dimers. So, dimers exhibiting an interface in the same region of the structure as Sfβgly (loop_2_, loop_3_-α-helix_3_, loop_4_-α-helix_4_ and loop_5_) were identified using the software PDBePISA to perform structural alignments. Eighteen GH1 β-glucosidases dimers, from Bacteria, Archaea and Eukaryota, which exhibit the highest degree of similarity with Sfβgly were selected to produce a multiple sequence alignment (MSA) (Figure S4). No highly conserved residue is observed in the interface. But uncharged and charged polar residues are the main occurrence in the positions involved in hydrogen bonds in the center and the edge of the interface, while apolar and polar residues are found in the positions participating in the hydrophobic effect.

### Effects of Mutation on the Enzymatic Activity

Finally, considering that dimerization affects the Sfβgly catalytic activity, the mutational effect on the enzyme kinetic parameters was evaluated (Table 3).

**Table 3:** Enzyme Kinetics Parameters for the Wild-Type and Mutant Sfβgly

Sfβgly	*k*_cat_ (min^–1^)	*K*_m_ (mM)	*k*_cat_/*K*_m_ (min^–1^ mM^–1^)	relative *k*_cat_/*K*_m_ (%)
wild-type	143 ± 2	1.1 ± 0.1	130 ± 14	100
N112S	140 ± 4	1.2 ± 0.2	117 ± 23	90 ± 27
N157S*	66 ± 2	1.1 ± 0.2	60 ± 13	46 ± 14
D166S*	80 ± 1	2.2 ± 0.2	35 ± 4	28 ± 7
M210A	116 ± 2	1.1 ± 0.2	105 ± 21	81 ± 24
L214A	167 ± 7	1.9 ± 0.4	84 ± 21	65 ± 23
Y303A	73 ± 3	2.6 ± 0.5	27 ± 7	22 ± 7

The *v*_0_ versus [S] plots are shown in the Figure S5. The substrate is *p*-nitrophenyl β-glucoside (NPβglc). The asterisk indicates mutations of residues that were involved in hydrogen bonds between the monomers in the interface of the wild-type protein dimer. Standard deviations were estimated based on three different rate determinations. See more details in the Materials and Methods.

As observed based on the relative *k*_cat_/*K*_m_, mutations D166S and Y303A resulted in reduced activity, whereas the remaining mutants had an activity similar to the wild-type enzyme (Table 3). Considering that, circular dichroism spectra showed that these mutant Sfβgly are folded (Figure S3), the relative *k*_cat_/*K*_m_ suggests that D166S and Y303A mutants had some subtle alteration in their spatial structure. On the other hand, the unaltered relative *k*_cat_/*K*_m_ of the N112S, N157S, M210A and L214A mutants is expected since their increased *K*_D_ (Table 2) and low concentration in the enzymatic assay (0.09 μM) dictate that they are monomers like the wild-type enzyme.

## Discussion

The crystallographic asymmetric unit does not necessarily reflect the biological oligomerization state of a protein, so a crucial task is to distinguish biologically significant protein interfaces from nonbiological interfaces in a crystal.^29,30^ PDBePISA server, which provides accurate predictions in over 80% of cases,^1^ was used to estimate the oligomerization state of 88 GH1 β-glucosidases crystallographic structures. Such analysis showed that 47% of those enzymes are monomeric in solution, while 30% are dimers, 2% are trimers, 14% are tetramers, 5% are hexamers, and 2% are octamers. These data are consistent with previous arguments that at least 50% of hydrolases form oligomers.^14^ Such oligomer predominance reinforces the relevance of the detection and characterization of the Sfβgly homodimerization in solution, under biologically relevant conditions.

Some proteins are predominantly found in their oligomeric state, exhibiting *K*_D_ in the nanomolar range.^8^ In contrast, other proteins exhibit a weaker tendency to associate and are classified as transient. In this case, they have higher *K*_D_, ranging from micromolar to millimolar.^31,32^ Regarding the analysis of the Sfβgly dimerization at different concentrations of the phosphate buffer, the *K*_D_ was shown to vary between 7 to 135 μM, depending on the ionic concentration.

The literature refers to those dimers, whose *K*_D_ ranges from micromolar to millimolar scale, as weak dimers and also states that most of them bury between 1200 to 2000 Å^2^ at their interfaces,^33^ recapping that the dimeric interface of Sfβgly buries 905 Å^2^. A smaller interface could imply fewer noncovalent interactions and a smaller contribution of the hydrophobic effect,^31^ which probably explains the low stability of Sfβgly homodimers.

Interfacial residues N112, M210, L214, and Y303 contribute only a small fraction of the solvent-accessible surface area of the total buried interface within each monomer (9, 3, 5, and 4%, respectively). However, it should be considered that their replacement with smaller residues not only removed the surface area at those restricted points, but also exposed the surface of the residues with which they interact at the interface. N112 interacts with Y303, which was also one of the mutated residues. Additionally, M210 contacts M110, and L214 interacts with L159 and W163. Together, the mutated residues and their interacting counterparts account for 16, 12, and 19% of the buried surface area of the interface within each monomer, respectively. Additionally, it should be noted that these pairs are duplicated at the interface, so each one corresponds to approximately 30% of the solvent-excluded surface area of the interface (32, 24, and 38%, respectively).

In summary, these sets of residues involved in the hydrophobic effect at the interface contribute similarly, with approximately 14 kJ/mol each (Table 2), to the affinity between the monomers of the Sfβgly dimer. These experimental values are close to those expected based on the contribution of 54.4 J/mol per Å^2^ of buried apolar surface area at protein interfaces,^34^ which would correspond to 15, 11, and 18 kJ/mol for N112—Y303, M210—M110, and L214—L159/W163, respectively.

As these residue sets correspond to approximately 30% of the solvent-accessible surface area buried in the dimer, but they account for 50% of the affinity between monomers, the removal of about one-third of the interface surface area from the solvent appears to be sufficient to stabilize the interaction between monomers, compelling them to the association. It also reinforces the hypothesis, based on the ionic concentration effect on the *K*_D_ (Figure 4), that the hydrophobic effect is crucial for stabilizing the Sfβgly homodimer.

Remarkably the contribution from the hydrophobic effect of just one of the residues mentioned above (N112, M210, L214, and Y303; ∼14 kJ/mol) is similar to the contribution of the complete set of the interfacial hydrogen bonds to the stabilization of the Sfβgly homodimer, which is 19 kJ/mol (Table 2). This observation points to a specific role for the hydrogen bonds. Indeed, they are distributed over the interface in a suggestive pattern that likely ensures specificity in the protein–protein interaction (Figure 6).

Indeed, as commented above, slightly more than 30% of the interface surface of each monomer interacting via the hydrophobic effect suffices for an inevitable dimerization. It would be precisely here, appreciating that the monomers have plenty of apolar interface area for misplaced binding, that hydrogen bond-forming residues might be critical for ensuring specificity, preventing the monomers from binding in an unviable orientation.

The position of hydrogen bonds at the interface and the corresponding mutational effect of their perturbation provide additional support to this hypothesis. The D166S mutation reduces the monomer affinity to 6.6 kJ/mol, while the N157S mutation results in a decrease of 2.9 *k*J/mol. This difference may be related to the positioning of these residues within the dimer interface. The D166 residues are located at both extremities of the interface, while the N157 residues are situated more centrally. So, the hydrogen bonds at the extremities, between D166 and W305, could play a more important role in maintaining homodimer stability. Disrupting these bonds at the interface extremity would have a greater impact on stability compared to disrupting hydrogen bonds near the center because the wobbly edges would expose hydrophobic patches to the solvent. In short, we conjecture that hydrogen bonds involving D166 and W305 would seal the interface edges.

These observations about the hydrogen bond distribution and the dimer instability when more than 30% of the interface becomes exposed to the solvent can be combined in a hypothetical picture of the homodimer assembly and disassembling. Thus, the dimerization would involve two Sfβgly monomers approaching due to the hydrophobic effect. When more than 30% of their interface surfaces were hidden from the solvent, the homodimer would be stable. However, in this supposed model, the ligation would progress up to the point that D166 and N157 were properly aligned for the hydrogen bond formation in the center of the interface. Such an anchorage point would be reinforced by the burial of the hydrophobic residues around it. Ultimately, the hydrogen bonds in the interface extremities, involving D166 and W305, could zip the association. Moving on, the continuous dynamics of the homodimer would eventually lead to a temporary separation at the peripheries of the interface, i.e., the hydrogen bond between D166 and W305 could be weakened or ruptured. Such a temporary departure would hypothetically open a breach to the water getting in. If that gap advanced beyond 70% of the interface surface then the dimer would collapse.

Finally, taking into account only the interface residues which role in dimerization was proposed here, the trends observed in the MSA (Figure S4) were: (1) hydrogen bond forming residues of the interface, which apparently confer the proper orientation to the monomer binding, tend to be conserved. Hence, the position corresponding to N157 is occupied by N, D, E, Q, T and S in 48% of the proteins, the positions 166 is occupied by D, E and N in 53% and finally the position 305 is occupied by W, D, E, S, K, R in 88%. Apolar residues at those positions are present in only 23, 5 and 12% of the β-glucosidases, respectively. (2) The hydrophobicity of the positions M210 and L214, which are relevant to propel the Sfβgly dimerization, is a minoritarian property among those GH1 β-glucosidases since apolar residues in those positions combine to only 15 and 39%, respectively. Indeed, polar residues, which could be involved in hydrogen bonds or ionic interactions, as T, S, N, E, Q, R and K, are more common in the 210 and 214 positions, occurring respectively in 85 and 71% of the dimers. (3) The residue Y303, which removal abolished the dimerization, is positioned in a loop that is present only in the insect GH1 β-glucosidase.

In conclusion, the combination of these trends suggests that the GH1 β-glucosidases dimerization interface presents two broad types regarding the molecular principles guiding the monomer binding: (1) dimers that rely on the hydrophobic effect, like Sfβgly, and (2) dimers in which polar interactions are probably the main force of the monomers binding. Regardless the type, in both groups the hydrogen bonds in the center and the edge of the interface seems to be important, so polar residues are conserved at those positions.

## Materials and Methods

### Site-Directed Mutagenesis

Site-directed mutagenesis experiments were performed by using QuikChange Lightning Site-Directed Mutagenesis Kit (Agilent Technologies, Santa Clara, CA, USA) according to the manufacturer’s instructions. Mutagenic primers are described in the Table S1. The pET-46 EK-LIC vector (Merck Millipore, Billerica, MA) containing the inset coding for Sfβgly (pET46-Sfβgly) was available in our group^19^ and served as the template for producing the mutants.

### Expression and Purification of Recombinant Proteins in Bacteria

The WT Sfβgly and mutants were expressed and purified according to the method described previously.^20^ Following purification, the wild-type and mutant Sfβgly samples underwent buffer exchange using PD Minitrap G-25 columns (Cytiva, Marlborough, MA, USA). The final samples were stored in a 100 mM phosphate buffer pH 6 (P100) at 4 °C. Sample purity was evaluated through SDS-PAGE^35^ and protein concentration was determined using the bicinchoninic acid (BCA) assay.^36^

To perform SEC chromatography of wild-type Sfβgly in buffers other than P100 the stored samples were exchanged to 75 mM (P75), 50 mM (P50), 25 mM (P25), or 5 mM (P5) phosphate buffer pH 6 using PD Minitrap G-25 columns.

### Size Exclusion Chromatography and Determination of the Homodimer Dissociation Constant (*K*_D_)

The SEC was conducted with an ÄKTA FPLC (GE HealthCare) system. Two Superdex 200 HR10/300 GL (GE HealthCare) columns were coupled in tandem and equilibrated with appropriate phosphate buffer pH 6. The columns were placed on crushed ice (5 °C) to enhance peak resolution. In that condition, the system pressure stabilized at 1.1 MPa with a flow rate of 0.25 mL/min. The wild-type and mutant Sfβgly samples were loaded into the system with a 100 μL sample loop. Three independent experiments were performed with the wild-type protein in P100 buffer to determine the reproducibility of the experiment and the standard deviation of the *K*_D_. A single protein sample was used in the SEC in different buffers with the wild-type and mutant proteins. Data were collected and processed by the UNICORN software version 5.11 (GE HealthCare). The area of the chromatographic peaks was used to estimate the relative abundance of monomers and dimers, which combined with the total protein concentration, resulted in the monomer and dimer concentration. Those data were used in the *K*_D_ determination as follows. Assuming a simple equilibrium between dimer (D) and monomer (M) Sfβgly

M+M⇌D

the dissociation constant (*K*_D_) is expressed as

$$K_{D}=\frac{{\left[M\right]}^{2}}{\left[D\right]}$$

which can be rearranged to

$${\left[M\right]}^{2}=K_{D}\left[D\right]$$

indicating that a plot of [M]^2^ versus [D] should be a line and its slope would correspond to the *K*_D_

### SEC-MALS

To determine the molar mass of the peaks observed in wild-type Sfβgly samples in P100, a SEC system as described above was connected to a miniDAWN TREOS multiangle static light scattering (MALS) and an Optilab T-rEX detector (Wyatt Technology Corporation, Santa Barbara CA). Experimental conditions were the same as those previously described. Data were collected and analyzed with the ASTRA software version 7.1.1.3 (Wyatt Technologies).

### Enzyme Activity of Wild-Type and Mutant Sfβgly

The initial rate (*v*_0_) of hydrolysis of at least 10 different concentrations of the substrate *p*-nitrophenyl-β-Dglucopyranoside (NPβglc) prepared in P100 buffer was used to determine the enzyme kinetic parameters (*k*_cat_ and *K*_m_). The diluted enzyme (50 μL) was mixed with the substrate (50 μL) in a 96-well plate while on ice. The enzymatic reaction began when the assay plates were moved to a 30 °C warm bath. To determine the initial rate, reactions at each substrate concentration were performed at four time intervals (5, 10, 15, and 20 min) to monitor product accumulation as a function of time. The reactions were stopped by adding 0.5 M Na_2_CO_3_ (100 μL). Absorbance at 415 nm was measured using an Elx800 microplate reader (Biotek) and converted to moles of the released product (4-nitrophenol) using a linear calibration curve. The product (in nmol) was plotted against the respective time intervals (5, 10, 15, and 20 min), and the *v*_0_ (nmol/min) was determined from the slope of the linear plots. The substrate concentration and their corresponding *v*_0_ were fitted to the Michaelis–Menten equation by using the software Origin 2019 to determine the kinetic parameters (*K*_m_, *k*_cat_, and *k*_cat_/*K*_m_). These assays for kinetic parameter determination were repeated three independently times.

### Structural and Sequence Alignment of the GH1 β-Glucosidase Dimers

The software PDBePISA was used was used to localize the dimer interface between monomers A and B in the Sfβgly structure (PDB 5CG0). Next the interface search automatic tool was employed to identify structurally similar interfaces among proteins in the PDB. Only GH1 β-glucosidase dimers exhibiting favorable binding energy (negative Δ*G*_i_) and significant similarity index (*Q* ≥ 0.5) were selected to the multiple sequence alignment. Redundant dimers, corresponding to different entries in the PDB for the same protein, were not considered. Finally, the sequence alignment was performed in the Clustal Omega automated tool at the Uniprot portal.

## Supplementary Material



## Notes

Additional results and materials details (PDF)

## Abbreviation

Sfβgly: GH1 β-glucosidase from *Spodoptera frugiperda* (PDB 5CG0)
