# Supplementary material for: Impact of Changes in Buffer Ionic Concentration and Mutations on a GH1 β‑Glucosidase Homodimer
Source: ACS Omega. 2025 Aug 7;10(32):35965–74. doi: 10.1021/acsomega.5c03396 (PMC12368724; doi:10.1021/acsomega.5c03396)
Supplement: Supplementary file 1 [file ao5c03396_si_001.pdf]

SUPPORTING INFORMATION

Impact of changes in buffer ionic concentration and mutations on a GH1 β-glucosidase homodimer

Rafael S. Chagas, Sandro R. Marana*

Departamento de Bioquímica, Instituto de Química, Universidade de São Paulo, São Paulo, SP, Brazil

*Corresponding author. Sandro R. Marana; E-mail: srmarana@iq.usp.br; Phone: +55 11 30918339

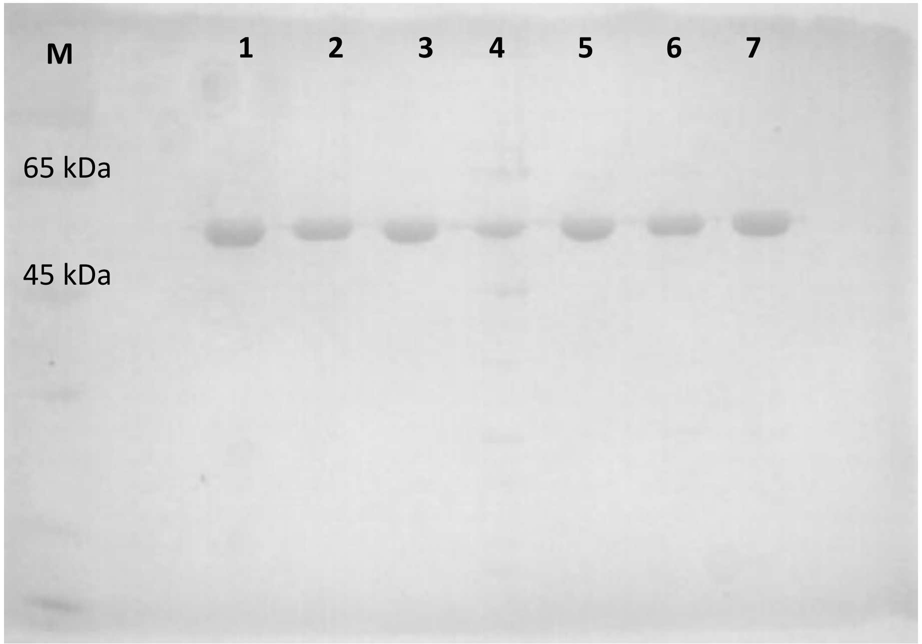

Figure S1 - SDS-PAGE of the purified recombinant wild-type and mutant Sfβgly. Lanes: **M** – Molecular weight marker (kDa). **1** – Wild-type Sfβgly; Mutant Sfβgly are: **2** – N112S; **3** – N157S; **4** – D166S; **5** – M210A; **6** – L214A; **7** – Y303A.

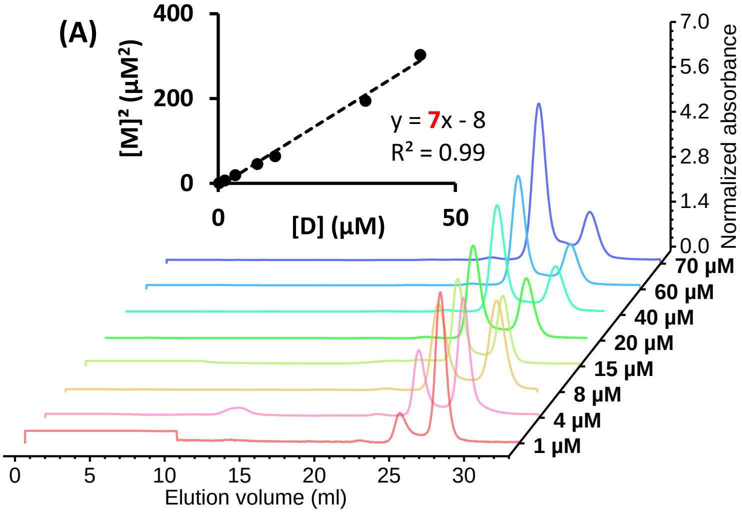

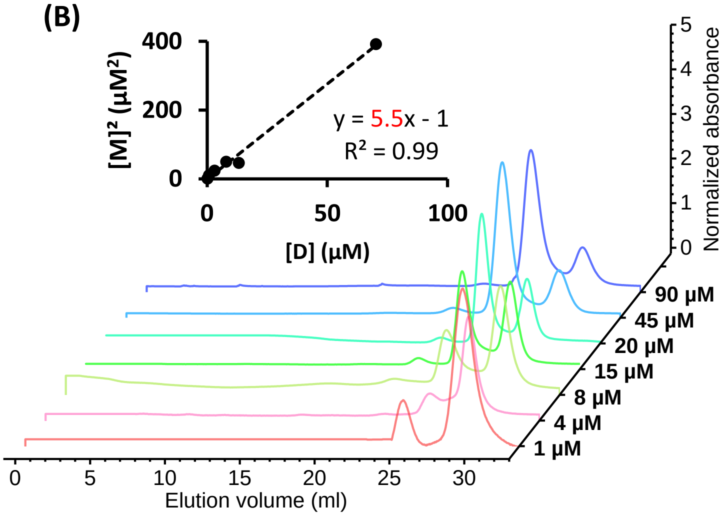

Figure S2 – Determination of the dissociation constant (K_D) of the Sfβgly dimer in two independent experiments. **A)** Protein sample 2; **B)** Protein sample 3. Both experiments (A and B) were performed with P100 buffer. [M] stands for monomer concentration. [D] is the homodimer concentration. Line slopes, which correspond to the K_D, are shown in red. Experiments were conducted at pH 6 at 5°C.

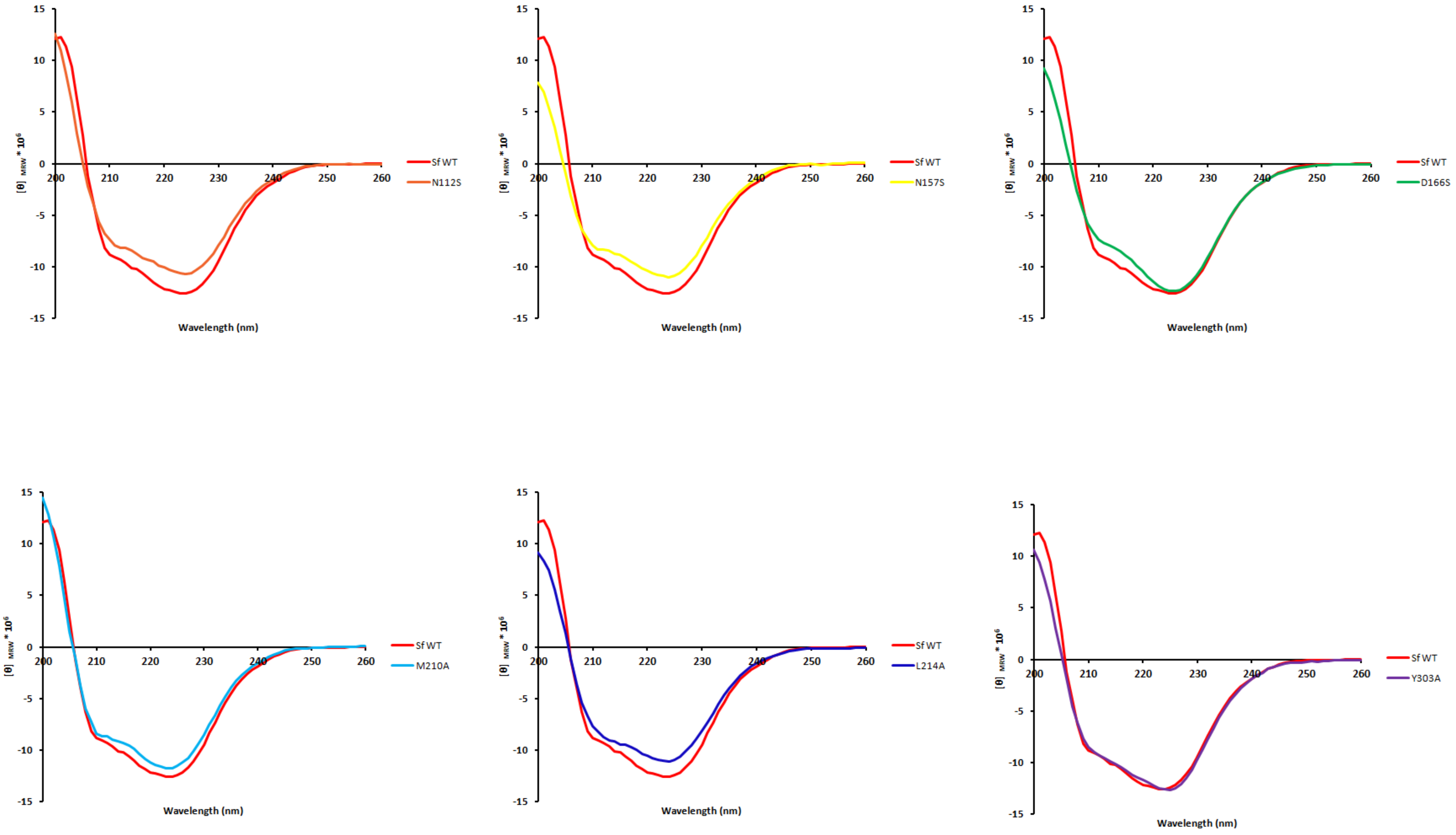

Figure S3 - Circular dichroism spectra of the wild-type and mutant Sfβgly. Spectra of the wild-type enzyme is in red and labelled SfWT, whereas the data of the mutant enzymes are presented in different colors and labeled with the simple mutation that they contain.

Figure S4 – Multiple sequence alignment of GH1 β-glucosidase dimers. Residues present in the dimerization interface, as previously identified in the Sfβgly crystallographic structure (5CG0_Spodoptera frugiperda; yellow background) by using the software PDBePISA, are in bold red font and marked with red boxes.

```
1UWQ_SULFOLOBUS_SOLFATARICUS          ------------------------------------------------------------     0
4HA4Acidilobus_saccharovorans         ------------------------------------------------------------     0
1QVB_Thermosphaera_aggregans          ------------------------------------------------------------     0
3APG_Pyrococcus_furiosus              ------------------------------------------------------------     0
5OKQ_Geobacillus_stearothermophilus   ------------------------------------------------------------     0
4IPL_Streptococcus_pneumoniae         --------------------------------------------------------HHHH     4
6WGD_Bacillus_licheniformis           ------------------------------------------------------------     0
2XHY_ESCHERICHIA_COLI                 ------------------------------------------------------------     0
4GZE_Lactobacillus_plantarum          ------------------------------------------------------------     0
4B3K_STREPTOCOCCUS_PYOGENES           ------------------------------------------------------------     0
5CG0_Spodoptera_frugiperda            ------------------------------------------------MKL---LVVLSL     9
3VIN_Neotermes_koshunensis            ------------------------------------------------------------     0
1WCG_BREVICORYNE_BRASSICAE            ------------------------------------------------------------     0
1CBG_Trifolium_repens                 -----------------------------------------------FKPLPI-------     6
1E1E_ZEA_MAYS                         ----------------------------------------------------SARVGSQN     8
1V03_SORGHUM_BICOLOR                  MALLLASAINHTAHPAGLRSHPNNESFSRHHLCSSPQNISKRRSNLSFRPRAQTISSESA     60
5BWF_Trichoderma                      ------------------------------------------------MGSSHHHHHHSS     12
1OIF_THERMOTOGA_MARITIMA              ------------------------------------------------MGSSHHHHHHSS     12
5DT5_Exiguobacterium_antarcticum      ------------------------------------------------MGSSHHHHHHSS     12


1UWQ_SULFOLOBUS_SOLFATARICUS          ------------MYSFPNSFRFGWSQAGFQSEMGTPGSEDPNTDWYKWVHDPENMAAG--     46
4HA4Acidilobus_saccharovorans         ------------AVTFPKDFLFGWSQAGFQSEMGTPGSEDPNSDWYAWVHDRENIAAG--     46
1QVB_Thermosphaera_aggregans          -------------MKFPKDFMIGYSSSPFQFEAGIPGSEDPNSDWWVWVHDPENTAAG--     45
3APG_Pyrococcus_furiosus              ------------MAKFPKNFMFGYSWSGFQFEMGLPGSE-VESDWWVWVHDKENIASG--     45
5OKQ_Geobacillus_stearothermophilus   MIHHHHHHEHRHLKPFPPEFLWGAASAAYQVEGAWNEDGKGLSVWDVFAKQPGRTFKGT-     59
4IPL_Streptococcus_pneumoniae         HHSSGLVPRGSHMTIFPDDFLWGGAVAANQVEGAYNEDGKGLSVQDVLPKGGLGEATENP     64
6WGD_Bacillus_licheniformis           --------MTEQTKKFPEGFLWGGAVAANQVEGAYNVGGKGLSTADVSPNGVMYPFDESM     52
2XHY_ESCHERICHIA_COLI                 --------MIVKKLTLPKDFLWGGAVAAHQVEGGWNKGGKGPSICDVLTGGAHGVPREIT     52
4GZE_Lactobacillus_plantarum          -----SNAMTIKGRAFPEGFLWGGAVAAHQLEGGYKEGGKGLSTADIMTLGTNERPREIT     55
4B3K_STREPTOCOCCUS_PYOGENES           -------------LAFPKEFWWGGATSGPQSEGRFAKQH--RNLFDYWYEEEPDLFYD--     43
5CG0_Spodoptera_frugiperda            VAVACNASIVRQQRRFPDDFLFGTATASYQIEGAWDEDGKGENIWDYMVHNTPEVIRD--     67
3VIN_Neotermes_koshunensis            ----MDVASSDTVYTFPDEFKLGAATASYQIEGAWDENGKGPNIWDTLTHEHPDYVVD--     54
1WCG_BREVICORYNE_BRASSICAE            -----------MDYKFPKDFMFGTSTASYQIEGGWNEDGKGENIWDRLVHTSPEVIKD--     47
1CBG_Trifolium_repens                 ---SFDDFSDLNRSCFAPGFVFGTASSAFQYEGAAFEDGKGPSIWDTFTHKYPEKIKD--     61
1E1E_ZEA_MAYS                         GVQMLSPSEIPQRDWFPSDFTFGAATSAYQIEGAWNEDGKGESNWDHFCHNHPERILD--     66
1V03_SORGHUM_BICOLOR                  GIHRLSPWEIPRRDWFPPSFLFGAATSAYQIEGAWNEDGKGPSTWDHFCHNFPEWIVD--     118
5BWF_Trichoderma                      GLVPRGSHML------PKDFQWGFATAAYQIEGAIDKDGRGPSIWDTFCAI-PGKIAD--     63
1OIF_THERMOTOGA_MARITIMA              GLVPRGSHMASNVKKFPEGFLWGVATASYQIEGSPLADGAGMSIWHTFSHT-PGNVKN--     69
5DT5_Exiguobacterium_antarcticum      GLVPRGSHMAS--MKFAPNFVFGTATSSYQIEGAHDEGGRTPSIWDTFCDT-DGKVFE--     67
                                                         *  * : :  * *          .


1UWQ_SULFOLOBUS_SOLFATARICUS          --LVS--GDLPENGPGYWGNYKTFHDNAQKMGLKIARLNVEWSRIFPNPLPRPQNF-DES     101
4HA4Acidilobus_saccharovorans         --LVS--GDFPENGPGYWGNYRKFHDAAQAMGLTAARIGVEWSRIFPRPTFDVKVDAEVK     102
1QVB_Thermosphaera_aggregans          --LVS--GDFPENGPGYWNLNQNDHDLAEKLGVNTIRVGVEWSRIFPKPTFNVKVPVERD     101
3APG_Pyrococcus_furiosus              --LVS--GDLPENGPAYWHLYKQDHDIAEKLGMDCIRGGIEWARIFPKPTFDVKVDVEKD     101
5OKQ_Geobacillus_stearothermophilus   ---------NGDVAVDHYHRYQEDVALMAEMGLKAYRFSVSWSRVFPDGN----------     100
4IPL_Streptococcus_pneumoniae         T-----EDNLKLIGIDFYHKYKEDISLFSEMGFNVFRTSIAWSRIFPKGD----------     109
6WGD_Bacillus_licheniformis           E-----SLNLYHEGIDFYHRYKEDIALFAEMGFKAFRTSIAWTRIFPNGD----------     97
2XHY_ESCHERICHIA_COLI                 KEVLPGKYYPNHEAVDFYGHYKEDIKLFAEMGFKCFRTSIAWTRIFPKGD----------     102
4GZE_Lactobacillus_plantarum          DGVVAGKYYPNHQAIDFYHRYPEDIELFAEMGFKCFRTSIAWTRIFPNGD----------     105
4B3K_STREPTOCOCCUS_PYOGENES           -------YVGPDTASDAYHQIESDLTLLASLGHNSYRTSIQWTRLIDDFE----------     86
5CG0_Spodoptera_frugiperda            -------LSNGDIAADSYHNYKRDVEMMRELGLDAYRFSLSWARILPTGM----------     110
3VIN_Neotermes_koshunensis            -------GATGDIADDSYHLYKEDVKILKELGAQVYRFSISWARVLPEGH----------     97
1WCG_BREVICORYNE_BRASSICAE            -------GTNGDIACDSYHKYKEDVAIIKDLNLKFYRFSISWARIAPSGV----------     90
1CBG_Trifolium_repens                 -------RTNGDVAIDEYHRYKEDIGIMKDMNLDAYRFSISWPRVLPKGKL---------     105
1E1E_ZEA_MAYS                         -------GSNSDIGANSYHMYKTDVRLLKEMGMDAYRFSISWPRILPKGTK---------     110
1V03_SORGHUM_BICOLOR                  -------RSNGDVAADSYHMYAEDVRLLKEMGMDAYRFSISWPRILPKGTL---------     162
5BWF_Trichoderma                      -------GTSGVTACDSYNRTAEDIALLKSLGAKSYRFSISWSRIIPKGGR---------     107
1OIF_THERMOTOGA_MARITIMA              -------GDTGDVACDHYNRWKEDIEIIEKLGVKAYRFSISWPRILPEGT----------     112
5DT5_Exiguobacterium_antarcticum      -------KHNGDVACDHYHRFEEDIQHIKQLGVDTYRFSIAWPRIFPS-K----------     109
                                              .   :            :.    * .: * *:
```

```
1UWQ_SULFOLOBUS_SOLFATARICUS            -KQDVTEVEINENELKRLDEYANKDALNHYREIFKDLKSRGLYFILNMYHWPLPLWLHDP    160
4HA4Acidilobus_saccharovorans           -GDDVLSVYVSEGALEQLDKMANRDAINHYREMFSDLRSRGITFILNLYHWPLPLWLHDP    161
1QVB_Thermosphaera_aggregans            ENGSIVHVDVDDKAVERLDELANKEAVNHYVEMYKDWVERGRKLILNLYHWPLPLWLHNP    161
3APG_Pyrococcus_furiosus                EEGNIISVDVPESTIKELEKIANMEALEHYRKIYSDWKERGKTFILNLYHWPLPLWIHDP    161
5OKQ_Geobacillus_stearothermophilus     ------------------GA-VNEKGLDFYDRLIEELRNHGIEPIVTLYHWDVPQALMDA    141
4IPL_Streptococcus_pneumoniae           ------------------EEEPNEAGLKYYDELFDELHAHGIEPLVTLSHYETPLYLARK    151
6WGD_Bacillus_licheniformis             ------------------ETEPNEEGLEFYDRLFDELLKYNIEPVVTISHYEMPLGLIKK    139
2XHY_ESCHERICHIA_COLI                   ------------------EAQPNEEGLKFYDDMFDELLKYNIEPVITLSHFEMPLHLVQQ    144
4GZE_Lactobacillus_plantarum            ------------------ESEPNEAGLQFYDDLFDECLKNGIQPVVTLAHFEMPYHLVKQ    147
4B3K_STREPTOCOCCUS_PYOGENES             ------------------QATINPDGLAYYNRVIDACLANGIRPVINLHHFDLPIALYQA    128
5CG0_Spodoptera_frugiperda              ------------------ANEVNPAGIAFYNNYIDEMLKYNITPLITLYHWDLPQKLQEL    152
3VIN_Neotermes_koshunensis              ------------------DNIVNQDGIDYYNNLINELLANGIEPMVTMYHWDLPQALQDL    139
1WCG_BREVICORYNE_BRASSICAE              ------------------MNSLEPKGIAYYNNLINELIKNDIIPLVTMYHWDLPQYLQDL    132
1CBG_Trifolium_repens                   ------------------SGGVNREGINYYNNLINEVLANGMQPYVTLFHWDVPQALEDE    147
1E1E_ZEA_MAYS                           ------------------EGGINPDGIKYYRNLINLLLENGIEPYVTIFHWDVPQALEEK    152
1V03_SORGHUM_BICOLOR                    ------------------AGGINEKGVEYYNKLIDLLLENGIEPYITIFHWDTPQALVEA    204
5BWF_Trichoderma                        ------------------DDPVNQLGIDHYAQFVDDLLEAGITPFITLFHWDLPEELHQR    149
1OIF_THERMOTOGA_MARITIMA                -------------------GRVNQKGLDFYNRIIDTLLEKGITPFVTIYHWDLPFALQLK    153
5DT5_Exiguobacterium_antarcticum        -------------------GQFNPEGMAFYKTLATRLQEEGIKPAVTLYHWDLPMWAHEE    150
                                                             :  .: .*         .    :.: *:  *

1UWQ_SULFOLOBUS_SOLFATARICUS            IRVRR-GDFTGPSGWLST---RTVYEFARFSAYIAWKFDDLVDEYSTMNEPNVVGGLGYV    216
4HA4Acidilobus_saccharovorans           IAIRR-GNLSAPSGWLDV---RTVIEFAKFSAYVAWKLDDLVYMYSTMNEPNVVWGLGYA    217
1QVB_Thermosphaera_aggregans            IMVRRMGPDRAPSGWLNE---ESVVEFAKYAAYIAWKMGELPVMWSTMNEPNVVYEQGYM    218
3APG_Pyrococcus_furiosus                IAVRKLGPDRAPAGWLDE---KTVVEFVKFAAFVAYHLDDLVDMWSTMNEPNVVYNQGYI    218
5OKQ_Geobacillus_stearothermophilus     -----------YGAWESR---RIIDDFDRYAVTLFQRFGDRVKYWVTLNEQNIFISFGYR    187
4IPL_Streptococcus_pneumoniae           -----------YHGWVDR---RMIHFYEKFARTVLERYKDKVKYWLTFNEVNSVLE----    193
6WGD_Bacillus_licheniformis             -----------YGGWKNR---KVIDCYEHYAKTVFTRYKEKVKYWMTFNEINMVLH----    181
2XHY_ESCHERICHIA_COLI                   -----------YGSWTNR---KVVDFFVRFAEVVFERYKHKVKYWMTFNEINNQRNWRAP    190
4GZE_Lactobacillus_plantarum            -----------YGGWRNR---KLIQFYLNFAKVCFERYRDKVTYWMTFNEINNQTNFESD    193
4B3K_STREPTOCOCCUS_PYOGENES             -----------YGGWESK---HVVDLFVAFSKVCFEQFGDRVKDWFVHNEPMVVVEGSYL    174
5CG0_Spodoptera_frugiperda              ------------GGFANP---LISDWFEDYARVVFENFGDRVKMFITFNEPREICFEGYG    197
3VIN_Neotermes_koshunensis              ------------GGWPNL---VLAKYSENYARVLFKNFGDRVKLWLTFNDPLTFMDGYAS    184
1WCG_BREVICORYNE_BRASSICAE              ------------GGWVNP---IMSDYFKEYARVLFTYFGDRVKWWITFNEPIAVCKGYSI    177
1CBG_Trifolium_repens                   -----------YRGFLGR---NIVDDFRDYAELCFKEFGDRVKHWITLNEPWGVSMNAYA    193
1E1E_ZEA_MAYS                           -----------YGGFLDKSHKSIVEDYTYFAKVCFDNFGDKVKNWLTFNEPQTFTSFSYG    201
1V03_SORGHUM_BICOLOR                    -----------YGGFLDE---RIIKDYTDFAKVCFEKFGKTVKNWLTFNDPETFCSVSYG    250
5BWF_Trichoderma                        -----------YGGLLNRT--EFPLDFENYARVMFKALP-KVRNWITFNEPLCSAIPGYG    195
1OIF_THERMOTOGA_MARITIMA                ------------GGWANRE--I-ADWFAEYSRVLFENFGDRVKNWITLNEPWVVAIVGHL    198
5DT5_Exiguobacterium_antarcticum        ------------GGWVNRD--S-VDWFLDFARVCFEELDGIVDSWITHNEPWCAGFLSYH    195
                                                    .  .         ::               :  . *:

1UWQ_SULFOLOBUS_SOLFATARICUS            -----GVKSGFPPGYLS------------FELSRRAMYNIIQAHARAYDGIKSVSK----    255
4HA4Acidilobus_saccharovorans           -----AVKSGFPPGYLC------------LECAGRAMKNLVQAHARAYDAVKAITK----    256
1QVB_Thermosphaera_aggregans            -----FVKGGFPPGYLS------------LEAADKARRNMIQAHARAYDNIKRFSK----    257
3APG_Pyrococcus_furiosus                -----NLRSGFPPGYLS------------FEAAEKAKFNLIQAHIGAYDAIKEYSE----    257
5OKQ_Geobacillus_stearothermophilus     -------LGLHPPGVKD------------MKRMYEANHIANLANAKVIQSFRHYVPD---    225
4IPL_Streptococcus_pneumoniae           -LPFTSGGIDIPKENLS------------KQELYQAIHHELVASSLVTKIAREINSE---    237
6WGD_Bacillus_licheniformis             -APFTGGGLVFEEGENK------------LNAMYQAAHHLFVASALAVKAGHDIIPD---    225
2XHY_ESCHERICHIA_COLI                   LFGYCCSGVVYTEHENP------------EETMYQVLHHQFVASALAVKAARRINPE---    235
4GZE_Lactobacillus_plantarum            GAMLTDSGIIHQPGENR------------ERWMYQAAHYELVASAAAVQLGHQINPD---    238
4B3K_STREPTOCOCCUS_PYOGENES             -------MQFHYPAIVD------------GKKAVQVAYNLALATAKVIQAYRRGPAELSD    215
5CG0_Spodoptera_frugiperda              -------SATKAPILNAT-----------AMGAYLCAKNLVTAHAKAYYLYDREFRPVQG    239
3VIN_Neotermes_koshunensis              -------EIGMAPSINTP-----------GIGDYLAAHTVIHAHARIYHLYDQEFRAEQG    226
1WCG_BREVICORYNE_BRASSICAE              -------K-AYAPNLNLK-----------TTGHYLAGHTQLIAHGKAYRLYEEMFKPTQN    218
1CBG_Trifolium_repens                   -------YGTFAPGRCSDWLKLNCTGGDSGREPYLAAHYQLLAHAAAARLYKTKYQASQN    246
1E1E_ZEA_MAYS                           -------TGVFAPGRCSPGLDCAYPTGNSLVEPYTAGHNILLAHAEAVDLYNKHYKR-DD    253
1V03_SORGHUM_BICOLOR                    -------TGVLAPGRCSPGVSCAVPTGNSLSEPYIVAHNLLRAHAETVDIYNKYHKG-AD    302
5BWF_Trichoderma                        -------SGTFAPGRQS------------TTEPWIVGHNLLVAHGRAVKVYRDEFKDLND    236
1OIF_THERMOTOGA_MARITIMA                -------YGVHAPGMRD------------IYVAFRAVHNLLRAHARAVKVFRETVKD---    236
5DT5_Exiguobacterium_antarcticum        -------LGQHAPGHTD------------MNEAVRAVHHMLLSHGKAVEMLKGEFNSA--    234
                                                                              :
```

```
1UWQ_SULFOLOBUS_SOLFATARICUS             KPVGIIYANSSFQPLTDK-----DMEAVEMAENDNRWWFFDAII--RGEITRGNEKIVR-      307
4HA4Acidilobus_saccharovorans            KPVGVIYANSDFTPLTDA-----DREAAERAKFDNRWAFFDAVV--RGQLGG----STR-      304
1QVB_Thermosphaera_aggregans             KPVGLIYAFQWFELLEGP-----AEVFDKF-KSSKLYYFTDIVS--KGSSI--INVEYR-      306
3APG_Pyrococcus_furiosus                 KSVGVIYAFAWHDPLAEE-----YKDEVEE-IRKKDYEFVTI------------------      293
5OKQ_Geobacillus_stearothermophilus      GKIGPSFAYSPMYPYDSR---PENVLAFENAEEFQNHWWMDVYA--WGMYPQAAWNYLES      280
4IPL_Streptococcus_pneumoniae            FKVGCMVLAMPAYPMTPN---PKDVWATHEYE-NLNYLFSDVHV--RGYYPNYAKRYFKE      291
6WGD_Bacillus_licheniformis              AKIGCMIAATTTYPMTPK---PEDVLAAMENE-RRTLFFSDVQA--RGAYPGYMKRFFKE      279
2XHY_ESCHERICHIA_COLI                    MKVGCMLAMVPLYPYSCN---PDDVMFAQESM-RERYVFTDVQL--RGYYPSYVLNEWER      289
4GZE_Lactobacillus_plantarum             FQIGCMIAMCPIYPLTAA---PADVLFAQRAM-QTRFYFADVHC--NGTYPQWLRNRFES      292
4B3K_STREPTOCOCCUS_PYOGENES              GRIGTILNLTPAYPASQS---EADMAAAHFAELWNNDLFMEAAV--HGKFPEELVAVLK-      269
5CG0_Spodoptera_frugiperda               GQCGITISVNWFGPATPT--P-EDEMAAELRRQGEWGIYAHPIFSAEGGFPKELSDKIAE      296
3VIN_Neotermes_koshunensis               GKVGISLNINWCEPATN---SAEDRASCENYQQFNLGLYAHPIFTEEGDYPAVLKDRVSR      283
1WCG_BREVICORYNE_BRASSICAE               GKISISISGVFFMPKNAE--SDDDIETAERANQFERGWFGHPVY--KGDYPPIMKKWVDQ      274
1CBG_Trifolium_repens                    GIIGITLVSHWFEPASKE---KADVDAAKRGLDFMLGWFMHPLT--KGRYPESMRYLVR-      300
1E1E_ZEA_MAYS                            TRIGLAFDVMGRVPYGTS---FLDKQAEERSWDINLGWFLEPVV--RGDYPFSMRSLAR-      307
1V03_SORGHUM_BICOLOR                     GRIGLALNVFGRVPYTNT---FLDQQAQERSMDKCLGWFLEPVV--RGDYPFSMRVSAR-      356
5BWF_Trichoderma                         GQIGIVLNGDFTYPWDSS-DPLD-REAAERRLEFFTAWYADPIY--LGDYPASMRKQLG-      291
1OIF_THERMOTOGA_MARITIMA                 GKIGIVFNNGYFEPASEKEEDIRAVRFMHQFNN--YPLFLNPIY--RGDYPELVLEFAR-      291
5DT5_Exiguobacterium_antarcticum         TPIGITLNLA---PKYAKTDSINDQIAMNNADGYANRWFLDPIF--KGQYPVDMMNLFS-      288
                                            .                                  :


1UWQ_SULFOLOBUS_SOLFATARICUS             ------------------------DDLKGRLDWIGVNYYTRTVVKRTEKGY-VSL-----      337
4HA4Acidilobus_saccharovorans            ------------------------DDLKGRLDWIGVNYYTRQVVRARGSGY-EIV-----      334
1QVB_Thermosphaera_aggregans             ------------------------RDLANRLDWLGVNYYSRLVYKIVDDKP-IIL-----      336
3APG_Pyrococcus_furiosus                 ------------------------LHSKGKLDWIGVNYYSRLVYGAKDGHL-VPL-----      323
5OKQ_Geobacillus_stearothermophilus      Q----G----LEPTV---APGDWELLQAAKPDFMGVNYYQTTTVEHNPPDG-VGEGVMNT      328
4IPL_Streptococcus_pneumoniae            N----D----INIEF---AAEDAELLKNYTVDFLSFSYYMSVTQSALPT-----------      329
6WGD_Bacillus_licheniformis              N----G----ITIEM---AEGDEDILKENTVDYIGFSYYMSMVASTSPE-----------      317
2XHY_ESCHERICHIA_COLI                    R----G----FNIKM---EDGDLDVLREGTCDYLGFSYYMTNAVKAEGG-----------      327
4GZE_Lactobacillus_plantarum             E----H----FNLDI---TAEDLKILQAGTVDYIGFSYYMSFTVKDTGK-----------      330
4B3K_STREPTOCOCCUS_PYOGENES              ---KDGVLWQSTPEE-------LALIAENRVDYLGLNFYHPKRVKAPDAIP-VISPSWSP      318
5CG0_Spodoptera_frugiperda               KSAQQGYPWSRLPEF---TEEEKAF-VRGTSDLIGVNHYTAFLVSATERKGPYPVPSLLD      352
3VIN_Neotermes_koshunensis               NSADEGYTDSRLPQF---TAEEVEY-IRGTHDFLGINFYTALLGKSGVEGY---EPSRYR      336
1WCG_BREVICORYNE_BRASSICAE               KSKEEGLPWSKLPKF---TKDEIKL-LKGTADFYALNHYSSRLVTFGSDPN----PNFNP      326
1CBG_Trifolium_repens                    ---------KRLPKF---STEESKE-LTGSFDFLGLNYYSSYYAAKAPRIP-NARPAIQT      346
1E1E_ZEA_MAYS                            ---------ERLPFF---KDEQKEK-LAGSYNMLGLNYYTSRFSKNIDISP-NYSPVLNT      353
1V03_SORGHUM_BICOLOR                     ---------DRVPYF---KEKEQEK-LVGSYDMIGINYYTSTFSKHIDLSP-NNSPVLNT      402
5BWF_Trichoderma                         ---------DRLPEF---TPEEKAF-VLGSNDFYGMNHYTSNYIRHRTSPA-TADDTVGN      337
1OIF_THERMOTOGA_MARITIMA                 ---------EYLPEN---YKDDMSE-IQEKIDFVGLNYYSGHLVKFDPDAP-AKV-----      332
5DT5_Exiguobacterium_antarcticum         ---------KYVHTYDFIHAGDLAT-ISTPCDFFGINFYSRNLVEFSAASD-FLH-----      332
                                                                        :  ....*


1UWQ_SULFOLOBUS_SOLFATARICUS             -------GGYGHGCERNSVSLAGLPTSDFGW-EFFPEGLYDVLTKYWNRYHL-YMYVTEN      388
4HA4Acidilobus_saccharovorans            -------PGYGHGCEPNGVSPAGRPCSDFGW-EFYPEGLYNVLKEYWDRYHL-PLLVTEN      385
1QVB_Thermosphaera_aggregans             -------HGYGFLCTPGGISPAENPCSDFGW-EVYPEGLYLLLKELYNRYGV-DLIVTEN      387
3APG_Pyrococcus_furiosus                 -------PGYGFMSERGGFAKSGRPASDFGW-EMYPEGLENLLKYLNNAYEL-PMIITEN      374
5OKQ_Geobacillus_stearothermophilus      TGKKGTSTSSGIPGLFKTVRNPHVDTTNWDW-AIDPVGLRIGLRRIANRYQL-PILITEN      386
4IPL_Streptococcus_pneumoniae            ------QYNSGEGNIIGGLVNPYLESSEWGW-QIDPIGLRIILNRYYDRYQI-PLFIVEN      381
6WGD_Bacillus_licheniformis              ------DLAKTEGNLLGGVKNPYLESSEWGW-QIDPKGIRITLNTLYDRYQK-PLFIVEN      369
2XHY_ESCHERICHIA_COLI                    -------TGDAISGFEGSVPNPYVKASDWGW-QIDPVGLRYALCELYERYQR-PLFIVEN      378
4GZE_Lactobacillus_plantarum             -------LA--YNEEHDLVKNPYVKASDWGW-QVDPVGLRYAMNWFTDRYHL-PLFIVEN      379
4B3K_STREPTOCOCCUS_PYOGENES              E------WYYDPYLMPGR-----RMNVDKGW-EIYPEAVYDIAIKMRDHYDNIPWFLSEN      366
5CG0_Spodoptera_frugiperda               D------VDTGS-----WADDSWLK-SASAWLTLAPNSIHTALTHLNNLYNKPVFYITEN      400
3VIN_Neotermes_koshunensis               D------SGVIL-----TQDAAWPI-SASSWLKVVPWGFRKELNWIKNEYNNPPVFITEN      384
1WCG_BREVICORYNE_BRASSICAE               D------ASYVT-----SVDEAWLKPNETPYIIPVPEGLRKLLIWLKNEYGNPQLLITEN      375
1CBG_Trifolium_repens                    D------SLIN--ATFEHNGKPLGPMAASSWLCIYPQGIRKLLLYVKNHYNNPVIYITEN      398
1E1E_ZEA_MAYS                            D------DAYASQEVNGPDGKPIGPPMGNPWIYMYPEGLKDLLMIMKNKYGNPPIYITEN      407
1V03_SORGHUM_BICOLOR                     D------DAYASQETKGPDGNAIGPPTGNAWINMYPKGLHDILMTMKNKYGNPPMYITEN      456
5BWF_Trichoderma                         V------DV----LFYNKEGQCIGPETESSWLRPCPAGFRDFLVWISKRYNYPKIYVTEN      387
1OIF_THERMOTOGA_MARITIMA                 -----------------SFVERDLPKTAMGW-EIVPEGIYWILKKVKEEYNPPEVYITEN      374
5DT5_Exiguobacterium_antarcticum         -----------------KDAYSDYDKTGMGW-DIAPSEFKDLIRRLAEYTDLPIYITEN      374
                                                                       :    *  .          *      : **
```

```
1UWQ_SULFOLOBUS_SOLFATARICUS              GIADDA-------------DYQRPYYLVSHVYQVHR-AINSGADVRGYLHWSLADNYEWA      434
4HA4Acidilobus_saccharovorans             GIADEG-------------DYQRPYYLVSHVYQVHR-ALQDGVNVIGYLHWSLADNYEWA      431
1QVB_Thermosphaera_aggregans              GVSDSR-------------DALRPAYLVSHVYSVWK-AANEGIPVKGYLHWSLTDNYEWA      433
3APG_Pyrococcus_furiosus                  GMADAA-------------DRYRPHYLVSHLKAVYN-AMKEGADVRGYLHWSLTDNYEWA      420
5OKQ_Geobacillus_stearothermophilus       GLGEFDTLEPG----DIVNDDYRIDYLRRHVQEIQRA-ITDGVDVLGYCAWSFTDLLSWL      441
4IPL_Streptococcus_pneumoniae             GLGAKDQLIKDELNNLTVQDDYRIQYMKEHLLQVAEA-LQDGVEIMGYTSWGCIDCVSMS      440
6WGD_Bacillus_licheniformis               GLGAVDVVEED----GSIQDDYRINYLRDHLKEVREA-IADGVDLIGYTSWGPIDLVSAS      424
2XHY_ESCHERICHIA_COLI                     GFGAYDKVEED----GSINDDYRIDYLRAHIEEMKKAVTYDGVDLMGYTPWGCIDCVSFT      434
4GZE_Lactobacillus_plantarum              GLGAIDKKTAD----NQIHDDYRIDYLTDHLRQIKLAVLEDGVDLIGYTPWGCIDLVAAS      435
4B3K_STREPTOCOCCUS_PYOGENES               GVGISGEDRY-RDETGQIQDDYRIQFLKEHLTYLH-KGIEAGSNCFGYHVWTPIDGWSWL      424
5CG0_Spodoptera_frugiperda                GWSTDES------RENSLIDDDRIQYYRASMESLLN-CLDDGINLKGYMAWSLMDNFEWM      453
3VIN_Neotermes_koshunensis                GFSDYG----------GLNDTGRVHYYTEHLKEMLKAIHEDGVNVIGYTAWSLMDNFEWL      434
1WCG_BREVICORYNE_BRASSICAE                GYGDDG----------QLDDFEKISYLKNYLNATLQAMYEDKCNVIGYTVWSLLDNFEWF      425
1CBG_Trifolium_repens                     GRNEFNDPT--LSLQESLLDTPRIDYYYRHLYYVL-TAIGDGVNVKGYFAWSLFDNMEWD      455
1E1E_ZEA_MAYS                             GIGDVDTKETPLPMEAALNDYKRLDYIQRHIATLK-ESIDLGSNVQGYFAWSLLDNFEWF      466
1V03_SORGHUM_BICOLOR                      GMGDIDKGDLPKP--VALEDHTRLDYIQRHLSVLK-QSIDLGADVRGYFAWSLLDNFEWS      513
5BWF_Trichoderma                          GTSLKGEND--LPKEKILEDDFRVNYYNEYIRAMFTAATLDGVNVKGYFAWSLMDNFEWA      445
1OIF_THERMOTOGA_MARITIMA                  GAAFDDV----VSEDGRVHDQNRIDYLKAHIGQAW-KAIQEGVPLKGYFVWSLLDNFEWA      429
5DT5_Exiguobacterium_antarcticum          GAAFDDQ----L-VDGKIHDQNRIDYVAQHLQAVS-DLNDEGMNIAGYLWSLLDNFEWS      428
                                          *                  *  :  :    :                **  *   *


1UWQ_SULFOLOBUS_SOLFATARICUS              SG-FSMRFGLLKVDYNTKR-----LYWRPSALVYREIATNGAITDEIEHLNSVPPVKPLR      488
4HA4Acidilobus_saccharovorans             SG-FSKRFGLLMVDYSTKR-----LHWRPSAFIYREIAKSRAITDEIEHLNSVPPLRGLS      485
1QVB_Thermosphaera_aggregans              QG-FRQKFGLVMVDFKTKK-----RYLRPSALVFREIATHNGIPDELQHLTLIQ------      481
3APG_Pyrococcus_furiosus                  QG-FRMRFGLVYVDFETKK-----RYLRPSALVFREIATQKEIPEELAHLADLKFVTRK-      473
5OKQ_Geobacillus_stearothermophilus       NG-YQKRYGFVYVNRDDESEKDLRRIKKKSFYWYQRVIETNGAEL---------------      485
4IPL_Streptococcus_pneumoniae             TAQLSKRYGLIYVDRNDDGSGTLNRYKKMSFTWYKEVIESNGESLFK-------------      487
6WGD_Bacillus_licheniformis               TAEMKKRYGYIYVDRDNEGKGTLSRTRKKSFYWYKKVIETNGESL---------------      469
2XHY_ESCHERICHIA_COLI                     TGQYSKRYGFIYVNKHDDGTGDMSRSRKKSFNWYKEVIASNGEKL---------------      479
4GZE_Lactobacillus_plantarum              TGQMSKRYGFIYVDENDDGSGSLKRYKKDSFTWFQHVIATNGAEIE--------------      481
4B3K_STREPTOCOCCUS_PYOGENES               NA-YKNRYGLVENNIHTQV-----RRPKASAYWFKKVATHNRLISLEVMEEFGGSASHLS      478
5CG0_Spodoptera_frugiperda                EG-YIERFGLYEVDFSDPA---RTRTPRKAAFVYKHIIKHRVVDYEYEPETMV---MTID      506
3VIN_Neotermes_koshunensis                RG-YSEKFGIYAVDFEDPA---RPRIPKESAKVLAEIMNTRKIPERFRDLEHH---HHHH      487
1WCG_BREVICORYNE_BRASSICAE                YG-YSIHFGLVKIDFNDPQ---RTRTKRESYTYFKNVVSTGKP-----------------      464
1CBG_Trifolium_repens                     SG-YTVRFGLVFVDFKNNL----KRHPKLSAHWFKSFLKK--------------------      490
1E1E_ZEA_MAYS                             AG-FTERYGIVYVDRNNNC----TRYMKESAKWLKEFNTAKKP--SKKILTPA-------      512
1V03_SORGHUM_BICOLOR                      SG-YTERFGIVYVDRENGC----ERTMKRSARWLQEFNGAAKKVENNKILTPA---GQLN      565
5BWF_Trichoderma                          DG-YVTRFGVTYVDYENGQ----QRFPKKSAKSLKPLFDELIA--KE-------------      485
1OIF_THERMOTOGA_MARITIMA                  EG-YSKRFGIVYVDYSTQK----RIV-KDSGYWY----SNVVK--NNGLED---------      468
5DT5_Exiguobacterium_antarcticum          FG-YDKRFGIIYVDFDTQE----RIW-KDSAHWY----ANVIQ--THKAALPQ---EA--      471
                                           .    ::*    :             : :


1UWQ_SULFOLOBUS_SOLFATARICUS              H---     489
4HA4Acidilobus_saccharovorans             PGHR     489
1QVB_Thermosphaera_aggregans              ----     481
3APG_Pyrococcus_furiosus                  ----     473
5OKQ_Geobacillus_stearothermophilus       ----     485
4IPL_Streptococcus_pneumoniae             ----     487
6WGD_Bacillus_licheniformis               ----     469
2XHY_ESCHERICHIA_COLI                     ----     479
4GZE_Lactobacillus_plantarum              ----     481
4B3K_STREPTOCOCCUS_PYOGENES               D---     479
5CG0_Spodoptera_frugiperda                EGH-     509
3VIN_Neotermes_koshunensis                ----     487
1WCG_BREVICORYNE_BRASSICAE                ----     464
1CBG_Trifolium_repens                     ----     490
1E1E_ZEA_MAYS                             ----     512
1V03_SORGHUM_BICOLOR                      ----     565
5BWF_Trichoderma                          ----     485
1OIF_THERMOTOGA_MARITIMA                  ----     468
5DT5_Exiguobacterium_antarcticum          ----     471
```

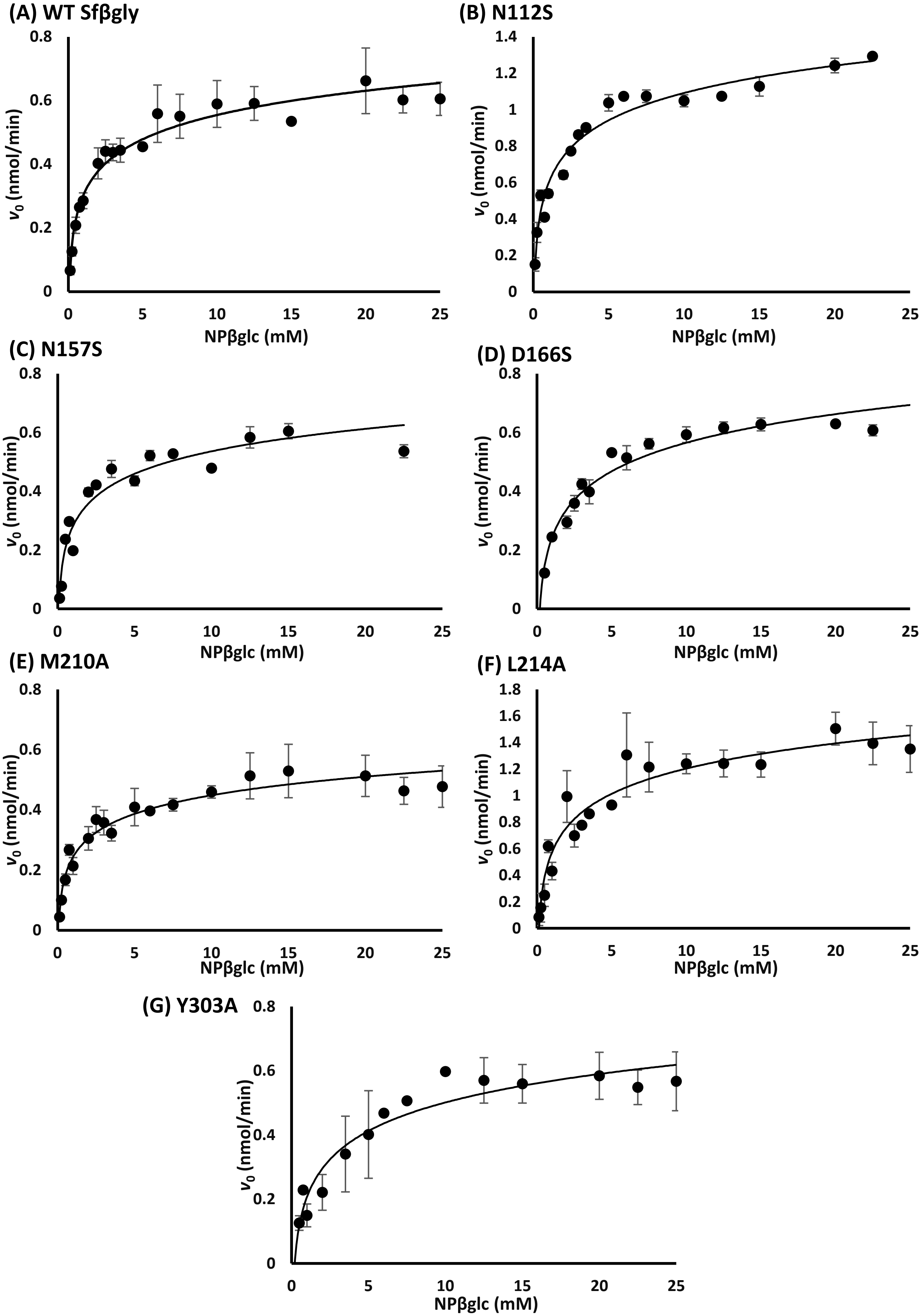

Figure S5 - Effect of NPβglc concentration on the activity of the wild-type and mutant Sfβgly. (**A**) wild-type Sfβgly, 0.0025 µg/µL; **(B)** N112S, 0.005 µg/µL; **(C)** N157S, 0.005 µg/µL; **(D)** D166S, 0.005 µg/µL; **(E)** M210A, 0.0025 µg/µL; **(F)** L214A, 0.005 µg/µL; **(G)** Y303A, 0.005 µg/µL.

Table S1 – Mutagenic primers employed to produce mutant Sfβgly.

Mutations	Pair of mutagenic primers
N112S	5'-tgggttgacctcactggccatgccagtgg-3'<br>5'-ccactggcatggccagtgaggtcaaccca-3'
N157S	5'-gatgaggggactggcgaaaccacccaactc-3'<br>5'-gagttgggtggtttcgccagtcccctcatc-3'
D166S	5'-gacaaccctcgcgtagctttcgaaccagtcgctg-3'<br>5'-cagcgactggttcgaaagctacgcgagggttgtc-3'
M210A	5'-cacacaagtaagctcccgccgcggtagcgtttagga-3'<br>5'-tcctaaacgctaccgcggcgggagcttacttgtgtg-3'
L214A	5'-ccaagttcttggcacacgcgtaagctcccatcgcgg-3'<br>5'-ccgcgatgggagcttacgcgtgtgccaagaacttgg-3'
Y303A	5'-gacgagaccaaggggcgccttgctgagcgc-3'<br>5'-gcgctcagcaaggcgccccttggtctcgtc-3'

Table S2 – GH1 β-glucosidases dimers presenting interface similar to Sfβgly.

Entry	Q score	Seq Id	Interface area (Å^2)	ΔG_i (kcal/mol)	CSS
3vin	0.863	0.495	943.9	-10.2	0.352
1wcg	0.791	0.468	1023.6	-17.9	0.612
1cbg	0.736	0.418	1354.0	-20.0	1,000
5bwf	0.715	0.400	1562.3	-11.7	0.237
1v03	0.699	0.395	1017.2	-12.7	0.240
1oif	0.696	0.405	1092.8	-12.3	1,000
1e1e	0.688	0.380	974.9	-11.7	1,000
4b3k	0.658	0.278	484.3	-5.8	0.608
5okq	0.647	0.319	1560.7	-8.4	0.439
5dt5	0.632	0.355	886.4	-10.2	0.612
6wgd	0.547	0.299	1512.9	-14.7	1,000
4ipl	0.520	0.277	1719.9	-12.3	0.905
2xhy	0.509	0.295	1792.8	-19.1	0.215
4ha4	0.508	0.293	1250.0	-13.2	1,000
1qvb	0.492	0.256	878.6	-8.5	1,000
1uwq	0.491	0.266	1314.1	-16.0	1,000
3apg	0.478	0.274	1197.4	-10.7	0.651
4gze	0.463	0.287	1592.0	-17.4	1,000

Entry, PDB identification code; Q score, similarity score based on rmsd and interface size evaluated based on a structural alignment between two crystallographic structures. Q ranges from 0 to 1; Seq Id, Sequence identity (%); ΔG_I, free energy of the monomer binding; CSS, complexation score based on the binding energy. It ranges from 0 to 1. Q and Seq Id were calculated in comparison to Sfβgly (5cg0).
